# Exploring the directionality of *Escherichia coli* formate hydrogenlyase: a membrane‐bound enzyme capable of fixing carbon dioxide to organic acid

**DOI:** 10.1002/mbo3.365

**Published:** 2016-05-02

**Authors:** Constanze Pinske, Frank Sargent

**Affiliations:** ^1^ Division of Molecular MicrobiologySchool of Life SciencesUniversity of DundeeDundeeScotlandDD1 5EHUnited Kingdom; ^2^ Present address: Martin‐Luther University Halle‐WittenbergInstitute of Biology/MicrobiologyKurt‐Mothes‐Str. 3, 06120Halle (Saale)Germany

**Keywords:** [NiFe] hydrogenase, bacterial hydrogen metabolism, formate chemosynthesis, formate dehydrogenase, formate hydrogenlyase, site‐directed mutagenesis

## Abstract

During mixed‐acid fermentation *Escherichia coli* produces formate, which is initially excreted out the cell. Accumulation of formate, and dropping extracellular pH, leads to biosynthesis of the formate hydrogenlyase (FHL) complex. FHL consists of membrane and soluble domains anchored within the inner membrane. The soluble domain comprises a [NiFe] hydrogenase and a formate dehydrogenase that link formate oxidation directly to proton reduction with the release of CO_2_ and H_2_. Thus, the function of FHL is to oxidize excess formate at low pH. FHL subunits share identity with subunits of the respiratory Complex I. In particular, the FHL membrane domain contains subunits (HycC and HycD) that are homologs of NuoL/M/N and NuoH, respectively, which have been implicated in proton translocation. In this work, strain engineering and new assays demonstrate unequivocally the nonphysiological reverse activity of FHL in vivo and in vitro. Harnessing FHL to reduce CO_2_ to formate is biotechnologically important. Moreover, assays for both possible FHL reactions provide opportunities to explore the bioenergetics using biochemical and genetic approaches. Comprehensive mutagenesis of *hycC* did not identify any single amino acid residues essential for FHL operation. However, the HycD E199, E201, and E203 residues were found to be critically important for FHL function.

## Introduction

In the absence of exogenous respiratory electron acceptors, the *γ*‐proteobacterium *Escherichia coli* is able to perform a mixed‐acid fermentation (Bettenbrock et al. 2014). Under these conditions glucose is metabolized to pyruvate by the Embden–Meyerhof–Parnas glycolytic pathway (Romano and Conway 1996), after which a range of organic acids, ethanol, CO_2_, and H_2_ are produced. This ability of *E. coli* to produce molecular hydrogen has been a continuous source of research interest (Pakes and Jollyman 1901; Stephenson and Stickland 1931), especially for its potential use as a renewable energy source (Rittmann and Herwig 2012).

The key to fermentative H_2_ production by *E. coli* is the formate hydrogenlyase (FHL) enzyme (Sauter et al. 1992), which catalyses the disproportionation of formate to hydrogen and carbon dioxide:

$$H{\text{CO}}_{2}^{-}+H^{+}↔{\text{CO}}_{2}+H_{2}$$

The ‘forward’ reaction (CO_2_ and H_2_ production from formate) is the only one observed under physiological fermentative conditions (Stephenson and Stickland 1932; Sawers et al. 1985), indeed expression and biosynthesis of active FHL are repressed until the correct environmental conditions prevail that will favor this reaction, that is, relatively high formate concentration and relatively low pH. The formate substrate (p*Ka *= 3.75) is produced endogenously by *E. coli* by the pyruvate formatelyase (PFL) enzyme, which utilizes oxygen‐sensitive radical chemistry to generate acetyl CoA and formate (Sawers and Watson 1998). While the acetyl CoA can be used to generate ATP *via* acetate kinase, the formate produced is initially secreted out of the cell and PFL interacts directly with a formate‐specific channel, FocA, in order to achieve this as efficiently as possible (Doberenz et al. 2014). However, under fermentative conditions, the secreted formate cannot be further respired and instead accumulates in the growth medium. This, together with a drop in the external pH caused by accumulation of several organic acids, triggers a reversal of the FocA formate channel function (Wang et al. 2009) and formate is taken back into the cell where it induces production of the FHL complex (Sawers 1994).

**Figure 1:**
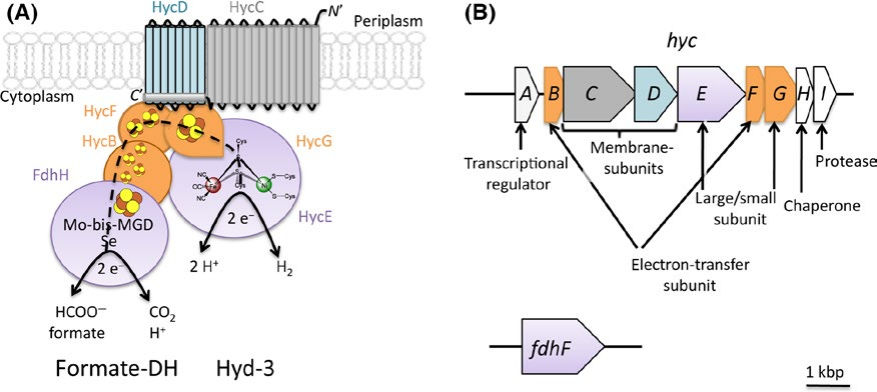
Schematic organization of the formate hydrogenlyase (FHL) proteins and the corresponding genes. (A) The FHL complex is oriented toward the cytoplasmic side of the membrane with the proteins HycD and HycC anchoring the complex (blue and gray, respectively) in the membrane, with small cylinders indicating the transmembrane helices. The [FeS]‐cluster harboring proteins HycB, HycF, and HycG are shown in orange, whereas the catalytic subunit of the formate dehydrogenase (formate‐DH, FdhF/FdhH protein) and the hydrogenase‐3 (Hyd‐3 and HycE protein) are shown in purple. The FDH‐H subunit contains a selenocysteine (Se) and a molybdopterin guanine dinucleotide (Mo‐bis‐MGD) at its active site, whereas Hyd‐3 harbors a [NiFe] cofactor. A dashed line indicates the predicted direction of electron flow. (B) The genes coding for FHL structural and accessory components are shown as arrows, with the colors corresponding to (A). The transcriptional regulator HycA, the chaperone HycH, and the HycE‐specific endoprotease HycI (all shown in white) are not part of the structural complex, whereas the *fdhF* gene (encoding the FDH‐H protein) is located on a different part of the chromosome. Modified from Pinske and Sawers (2014).

The *E. coli* FHL complex is a multimeric protein complex of seven subunits anchored at the cytoplasmic side of the membrane (Fig. 1) (Sauter et al. 1992; McDowall et al. 2014). The enzyme consists of a cytoplasmic domain of five subunits, including the products of the *hycB*,* hycE*,* hycF*,* hycG*, and *fdhF* genes, and a membrane domain made up of the *hycC* and *hycD* gene products (Fig. 1). The cytoplasmic domain contains a [NiFe]‐hydrogenase catalytic subunit (termed Hyd‐3) encoded by the *hycE* gene (Böhm et al. 1990). HycE contains a Ni‐Fe‐CO‐2CN^−^ cofactor, which is the site of proton reduction during H_2_ production (Peters et al. 2015), and this protein is linked by a molecular wire of Fe‐S clusters located within the HycG, HycF, and HycB proteins to a selenium‐ and molybdenum‐dependent formate dehydrogenase subunit encoded by the *fdhF* gene (Sauter et al. 1992; McDowall et al. 2014). The formate dehydrogenase component of FHL is commonly referred to as FDH‐H (formate dehydrogenase linked to hydrogen production) and it catalyses the oxidation of formate to CO_2_ (Khangulov et al. 1998). The cytoplasmic domain of the FHL complex therefore functions as a closed electron transfer system to directly connect two redox reactions.

FHL‐like complexes are conserved across the prokaryotic domains and the components share clear sequence identity with the energy‐conserving Complex I chain (Böhm et al. 1990; Efremov and Sazanov 2012). The FHL membrane domain contains two integral membrane proteins (HycC and HycD) that share common features with the NuoL/M/N and NuoH membrane proteins of Complex I that are directly involved in proton pumping (Brandt 2006; Efremov and Sazanov 2012; Batista et al. 2013). Moreover, analysis of the thermodynamics of the FHL forward reaction suggests that enough free energy could be generated to be coupled directly to proton translocation. Under standard conditions, the oxidation of formate (CO_2_/formate *E*_o_’ −432 mV) coupled to proton reduction (H^+^/H_2_ *E*_o_’ −414 mV) gives a small driving force (18 mV) that would in itself not be regarded as sufficient to translocate a proton (Thauer et al. 1977). However, FHL does not operate under standard conditions, instead it is expressed under certain physiological conditions that would lead to an increase in the available driving force for proton translocation (McDowall et al. 2014). Indeed, although *E. coli* cannot grow with formate as sole carbon and energy source, the archaeon *Thermococcus onnurineus* can grow using formate as electron donor (Kim et al. 2010; Lim et al. 2014). Here, the *T. onnurineus* FHL complex generates an initial proton gradient that is subsequently converted into a sodium gradient by a Na^+^/H^+^ antiporter, which can finally be coupled directly to Na^+^‐dependent ATP synthesis (Kim et al. 2010; Lim et al. 2014).

The possibility that FHL could be energy conserving has also led to hypotheses on the origins of life itself. One compelling idea is that the FHL ‘reverse’ reaction may once have dominated early cellular life, coupling the proton motive force to H_2_‐dependent CO_2_ fixation (Nitschke and Russell 2009). Certainly, the individual enzyme components of FHL have been shown to be reversible, as Hyd‐3 can link H_2_ oxidation to benzyl viologen or methyl viologen reduction in vitro and in situ (Krasna 1984; Sawers et al. 1985; Maeda et al. 2007). The isolated FDH‐H enzyme is bidirectional in electrochemistry experiments (Bassegoda et al. 2014). Moreover, very early work with intact wild‐type *E. coli* cells showed that formic acid could be produced when the cells were incubated under H_2_ and CO_2_ (Woods 1936). However, this experiment was done before modern biochemical and genetic experiments established that *E. coli* contains multiple formate dehydrogenases and hydrogenases that are reversible in vivo (Sawers 1994; Deplanche et al. 2010; Pinske et al. 2015). In addition, *E. coli* is now known to carry the genes for components of an alternative version of FHL (Andrews et al. 1997). Thus, the specific ability of *E. coli* FHL to perform the ‘reverse’ reaction deserves further investigation, not least as the harnessing of an alternative biological reaction capable of synthesizing organic acid from CO_2_ may have several biotechnological applications.

In this work, experimental evidence is provided that demonstrate *E. coli* FHL is reversible in vivo and also when isolated and assayed in vitro. This new ability to observe both the forward and reverse FHL reactions provided a means for characterization of the bioenergetics of *E. coli* FHL. In addition, a series of site‐directed mutagenesis experiments against the FHL membrane‐domain components HycC and HycD was undertaken. A total of 17 separate amino acid substitutions were made in HycC and none had a negative effect on FHL function. In the case of HycD, however, substitution of the conserved E138, E199, and E203 residues with alanine all resulted in reduced FHL activity, but as a result of incorrect assembly of the enzyme. Taken altogether, the data presented here do not support the hypothesis that *E. coli* FHL is energy conserving.

## Methods

### Strains, plasmids, and growth conditions

All bacterial strains and plasmids used in this study are listed in Tables 1 and 2. A marker‐less deletion of the genes for the membrane subunits *hycCD* was constructed by cloning the 500 bp upstream region as a HindIII/EcoRI fragment, and a 500 bp downstream region as a EcoRI/HindIII fragment, with the primers stated in Table S1, joining them together in pMAK705 before moving them to the chromosome of strain MG059e1 (Hamilton et al. 1989; McDowall et al. 2014). The chromosomal modifications were carefully constructed so as to preserve identifiable regulatory elements, coding sequences, stop codons, and ribosome‐binding sites of the flanking genes *hycB* and *hycE*.

**Table 1:** Forward and reverse in vivo formate hydrogenlyase (FHL) activities assessed in whole cells

Strain	Relevant genotype/exchange	Formate production (% of parental)^a^	H_2_ production (% of parental)^b^
RT1	Δ*hyaB, hybC, fdhE, pflA*	100 ± 23	118 ± 14^c^
RT2	Δ*hyaB, hybC, fdhE, pflA, hycAI*	13 ± 64	<1
JW2693	Δ*hycC*	18 ± 15	7 ± 53
JW2692	Δ*hycD*	16 ± 3	1 ± 1
EhisdCD	Δ*hycCD*	12 ± 34	<1
CP734	Δ*hyaB* Δ*hybC*	111 ± 11	100 ± 20
T83A	As CP734 HycC_T83A_	114 ± 18	106 ± 20
E135A	As CP734 HycC_E135A_	102 ± 12	101 ± 10
H222A	As CP734 HycC_H222A_	95 ± 21	101 ± 23
K239A	As CP734 HycC_K239A_	105 ± 15	80 ± 19
E281A	As CP734 HycC_E281A_	104 ± 20	96 ± 13
T292A	As CP734 HycC_T292A_	110 ± 17	106 ± 31
E294A	As CP734 HycC_E294A_	99 ± 17	103 ± 41
N295A	As CP734 HycC_N295A_	112 ± 20	96 ± 7
H328A	As CP734 HycC_H328A_	106 ± 21	112 ± 16
K336A	As CP734 HycC_K336A_	108 ± 14	110 ± 6
N386A	As CP734 HycC_N386A_	107 ± 24	100 ± 11
F388A	As CP734 HycC_F388A_	105 ± 10	104 ± 25

a Activity was calculated based on a single‐point assay following incubation of equal amounts of pregrown cells washed and suspended in 3‐mL 20 mmol/L MOPS (pH 7.4) in sealed Hungate tubes containing a H_2_ and CO_2_ atmosphere. A value of 100% corresponds to 5.1 mmol/L formate (final concentration), which is the amount produced by an FHL^+^ parental strain following 5 h incubation. The ± values indicate the percentage of the standard deviation from the respective value (*n* > 3).

b Reaction rates were calculated using a continuous assay of pregrown, washed, live cells in a H_2_‐sensing electrode. The reaction was started by the addition of excess formate and an initial rate of the parental strain CP734 was 37.8 ± 7.6 nmol H_2_ produced min^−1^ mg^−1^, and this value was used to correspond to 100% activity. The ± values indicate the percentage of the standard deviation from the respective value (*n* > 3).

c This value was obtained in the presence of an O_2_‐scavenging system as described (Sargent et al. 1999).

**Table 2:** No effect of chemical uncouplers on in vivo formate hydrogenlyase (FHL) activity

Addition	Effect	Formate production (% of parental)^a^	H_2_ production (% of parental)^b^
None	None	100 ± 23	100 ± 20
CCCP (100 *μ*mol/L)	H^+^	128 ± 21	58 ± 23
2,4‐dinitrophenol (100 *μ*mol/L)	H^+^	72 ± 11	100 ± 20
Monensin (20 *μ*mol/L)	Na^+^/H^+^	90 ± 2	79 ± 3
EIPA (50 *μ*mol/L)	Na^+^/H^+^	86 ± 10	79 ± 11
EIPA + Na^+^	Na^+^/H^+^	112 ± 8	67 ± 13
Na^+^ (112 mmol/L)	Na^+^	115 ± 2	62 ± 13
Nigericin (10 *μ*mol/L)	K^+^/H^+^	92 ± 4	133 ± 18
Valinomycin (10 *μ*mol/L)	K^+^	115 ± 14	95 ± 6
Valinomycin + K^+^	K^+^	121 ± 18	88 ± 10
K^+^ (112 mmol/L)	K^+^	106 ± 11	62 ± 22
Gramicidin (10 *μ*g mL^−1^)	K^+^	86 ± 8	107 ± 3
DCCD (35 *μ*mol/L)	ATPase	124 ± 29	107 ± 18
Crude extracts	No membrane potential	76 ± 9	12 ± 36
DMSO (28 mmol/L)	Solvent control	125 ± 25	110 ± 8

a This is a single end‐point assay of the amount of formate produced from a suspension of harvested, washed cells. Unless otherwise stated, strain RT1 (FHL^+^) was grown overnight anaerobically in TGYEP, pH 6.5, harvested, and washed in 20‐mmol/L MOPS buffer, pH 7.4, and resuspended in 3 mL of the same buffer before being incubated with H_2_ and CO_2_ in Hungate tubes for 5 h. The relative percentage was calculated based on the final concentration of formate produced by the RT1 parent strain being 5.1 ± 1.2 mmol/L being taken as 100%, with ± values indicating the percentage of the standard deviation from the respective value (*n* > 3).

b This is a reaction rate calculated using harvested and washed cells. Unless otherwise stated, cells of strain CP734 (FHL^+^) were grown overnight anaerobically in TGYEP, pH 6.5, harvested, and measured in a Clark‐type H_2_‐sensing electrode. The relative percentage was calculated based on a rate of the parental strain CP734 of 37.8 ± 7.6 nmol min^−1^ mg^−1^ being taken as 100%, with ± values indicating the percentage of the standard deviation from the respective value (*n* > 3).

Site‐directed mutations were introduced on a plasmid pKS‐hycCD, which is a derivative of pBluescriptKS+ containing the *hycCD* locus amplified using primers hycB‐500FW and hycE+500RW (Table S1) and cloned as a HindIII/HindIII fragment. Point mutations were generated using either the Q5 protocol (NEB) or Pfu (Stratagene method) and the primers used are listed in Table S1. After sequencing, the insert was moved into pMAK705 and subsequently moved to the chromosome (Hamilton et al. 1989) where the corresponding gene region was sequenced again.

Strains were routinely grown with shaking in LB medium or on agar plates (Miller 1972). Anaerobic growth was performed in TGYEP medium, pH 6.5 (Begg et al. 1977) in sealed bottles. The antibiotics ampicillin, chloramphenicol, and kanamycin were added to the medium at the final concentration of 100 *μ*g mL^−1^, 12.5 *μ*g mL^−1^, and 50 *μ*g mL^−1^, respectively.

### Determination of enzyme activities

Activity of the FHL complex was determined by formate‐dependent H_2_ production either in a qualitative way by monitoring gas production in Durham tubes after growth in LB + 0.8% (w/v) glucose (Guest 1969) or quantitatively using a modified Clark‐type electrode (Oxygraph) (Sargent et al. 1999). Briefly, an amount of 1.7‐mL anaerobic 20 mmol/L MOPS buffer, pH 7.0, was loaded into the electrode chamber before an aliquot of harvested and washed fermentatively grown cells was added. When the baseline was stable, formate was added to a final concentration of 15 mmol/L and the resulting slope used to calculate the H_2_ production rate, based on calibration with known amounts of H_2_‐saturated buffer (Sargent et al. 1999).

The Hyd‐3 and FDH‐H activities were determined as H_2_‐ or formate‐dependent BV reduction, respectively, as described (Pinske et al. 2011a).

High‐performance liquid chromatography (HPLC) analysis was performed using the Dionex UltiMate 3000 system equipped with an Aminex HPX‐87H column (BioRad, Hercules, California, USA). The isocratic eluent used was 5 mmol/L H_2_SO_4_ with a flow of 0.5 mL min^−1^ at 50°C for 30 min with UV detection at 210 nm. The formate peak eluted at 16.2 min and a standard curve was prepared between 1 and 200 nmol formate. Samples were prepared following anaerobic growth, after which the cells were washed with 20 mmol/L MOPS buffer, pH 7.4, and resuspended in that buffer, unless otherwise stated. An amount of 0.5‐mL cell suspension corresponding to approximately 25 mg of protein was added to Hungate tubes containing 2.5 mL of buffer and any additions as specified. The tubes were closed and H_2_ flushed through them for 5 min (12 mL). An amount of 5 mL CO_2_ gas was added to the tube, which corresponded to 29%. The cells were incubated at 37°C for 5 h, unless otherwise stated.

Ionophore experiments were done using carbonyl cyanide *m*‐chlorophenyl hydrazone (CCCP) prepared in dimethyl sulfoxide (DMSO) at a final concentration of 100 *μ*mol/L; 2,4‐dinitrophenol in H_2_O at 100 *μ*mol/L; monensin in ethanol at 20 *μ*mol/L; EIPA (5‐(*N*‐ethyl‐*N*‐isopropyl)‐amiloride) in DMSO at 50 *μ*mol/L; nigericin in DMSO at 10 *μ*mol/L; valinomycin in ethanol at 10 *μ*mol/L; gramicidin in ethanol at 10 *μ*g mL^−1^; and *N,N’*‐Dicyclohexylcarbodiimide (DCCD) in dimethyl formamide (DMF) at 35 *μ*mol/L. Where indicated, 112 mmol/L K^+^ or Na^+^ ions were present in the buffers. Crude extracts were prepared by sonication for 5 min before unbroken cells were removed by centrifugation.

### Substrate calculations

The concentrations of gases in the liquid phase was calculated according to Henry's law using the gas constants 7.8 × 10^−4^ mol L^−1^ atm^−1^ and 3.4 × 10^−2^ mol L^−1^ atm^−1^ for H_2_ and CO_2_, respectively. The partial pressures of each gas were calculated as fractions according to the ideal gas law.

### Protein purification and analysis

Purification of the FHL complex and subcomplex was done as described previously (McDowall et al. 2014). Samples were analyzed using 10% (w/v) SDS‐PAGE (Laemmli 1970) and either stained with InstantBlue (Expedeon, San Diego, California, USA) or transferred to nitrocellulose membranes as described (Towbin et al. 1979). Antibodies raised against HycG (1:3000 (Sauter et al. 1992)) were used. Secondary antibody conjugated to horseradish peroxidase was obtained from Bio‐Rad and visualization was done by enhanced chemiluminescence reaction (Stratagene, La Jolla, California, USA).

## Results

### Chemosynthesis of formate from CO_2_ in vivo driven by FHL

**Figure 2:**
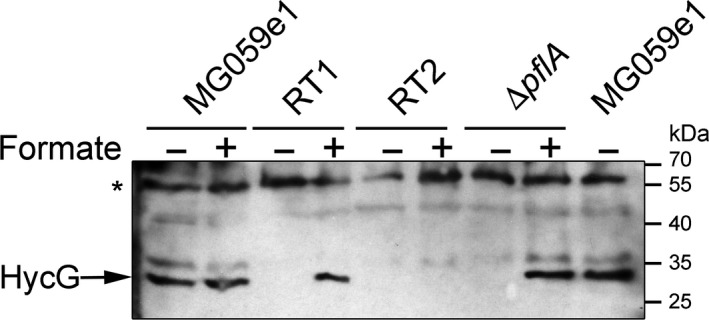
Production of formate hydrogenlyase (FHL) in response to exogenous formate. *E. coli* strains MG059e1 (*hycE* ^His^), RT1 (FHL ^+^), RT2 (FHL ^−^), and a Δ*pflA* control strain were grown anaerobically in TGYEP medium (pH 6.5) either in the presence or absence of 15 mmol/L formate as indicated. Following overnight growth at 37°C, cells were harvested, washed, and 25 *μ*g of total cell protein separated by SDS‐PAGE (10% (w/v) acrylamide). Protein was transferred to nitrocellulose and subsequently challenged with an antibody raised against *E. coli* HycG. The asterisk marks the position of a nonspecific cross‐reacting protein that serves as an internal loading control. Positions of the molecular weight markers (PageRuler, Fermentas) are indicated.

Early experiments using the chemical reduction of mercuric chloride as an assay for formic acid demonstrated that intact *E. coli* cells could generate formate from hydrogen and carbon dioxide (Woods 1936). However, this early work was unable to biochemically or genetically identify the enzymes responsible, and it therefore remained conceivable that multiple biochemical pathways were involved. To address this directly here, an *E. coli* strain (RT1) was constructed that was devoid of all hydrogenase and formate dehydrogenase activities save for FHL, and was also unable to produce formate endogenously through glycolysis. To achieve this, the *E. coli* MC4100 parental strain was engineered to carry a Δ*fdhE* allele, thus rendering it devoid of the respiratory formate dehydrogenases FDH‐O and FDH‐N (Schlindwein et al. 1990), and deletions of *hyaB* and *hybC*, thus removing the catalytic subunits of the respiratory [NiFe]‐hydrogenases Hyd‐1 and Hyd‐2. Finally, a deletion of the *pflA* gene, which codes for an enzyme required for activating the pyruvate formatelyases PflB and YfiD that generate endogenous formate in *E. coli* (Knappe et al. 1984; Wyborn et al. 2002), was included. Note that without production of endogenous formate the RT1 cells must be cultured in the presence of externally added formate in order to induce transcription of FHL‐coding genes. Formate‐dependent production of FHL in the RT1 strain was confirmed by Western analysis (Fig. 2). The Hyd‐3 small subunit, HycG, was only produced when the RT1 strain was cultured anaerobically in the presence of exogenous formate (Fig. 2).

**Figure 3:**
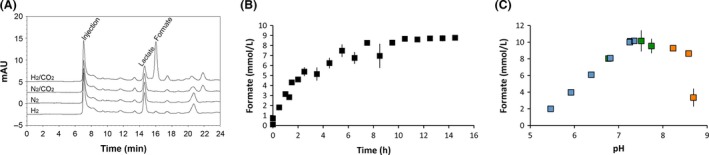
Characterization of the reverse formate hydrogenlyase (FHL) reaction in vivo. (A) The RT1 (Δ*hyaB hybC fdhE pflA* FHL ^+^) strain was grown overnight in TGYEP media, pH 6.5, supplemented with formate to induce expression of the formate regulon. The cells were harvested, washed, and 25 mg of cell protein incubated in 20 mmol/L MOPS (pH 7.4) for 8 h under 12 mL H_2_/5 mL CO_2_; 12 mL N_2_/5 mL CO_2_; N_2_ only; or H_2_ only, as indicated. An aliquot of the cell suspension was removed, cells removed by filtration, 10 *μ*L of the clarified supernatant applied to an Aminex HPX 87H column at 50°C, and separated compounds detected at A_210 nm_. (B) Time dependence of formate production. Cultures of strain RT1 were pregrown under FHL‐inducing conditions and washed cells corresponding to 25‐mg protein were incubated under a H_2_/CO_2_ atmosphere in 20 mmol/L MOPS (pH 7.4) and the final formate concentration in the aqueous phase of the reaction vessel was analyzed by high‐performance liquid chromatography (HPLC). (C) The pH optimum for formate production by intact cells. RT1 cells were pregrown under FHL‐inducing conditions before being washed and suspended in 200 mmol/L potassium phosphate buffer (*blue* data points); 200 mmol/L MOPS buffer (*green*); or 200 mmol/L boric acid buffer (*orange*) at the stated pH values that were determined after CO_2_ incubation. The final concentration of produced formate in the reaction vessels was quantified by HPLC after 5 h incubation. Each data point shows the mean values of three independent culture tubes with standard deviations.

Next, the RT1 (FHL^+^) strain was cultured in rich media under fermentative conditions in the presence of formate. This initial growth provided a stock of cells that had produced FHL components. Production of the FHL component HycG by RT1 under these conditions was confirmed by Western immunoblot analysis (Fig. 2). The FHL‐containing cells were harvested before being washed and suspended in 20 mmol/L MOPS buffer (pH 7.4) containing no other additions. An aliquot of cells (corresponding to 25 mg of total cell protein) was then incubated in a final volume of 3‐mL MOPS buffer at 37°C under a CO_2_ and H_2_ atmosphere (equivalent to 39 mmol/L H_2_ and 39 mmol/L CO_2_ in the gas phase and consequently 0.8 mmol/L H_2_ and approximately 37 mmol/L CO_2_ in the liquid phase at the beginning of the experiment). Next, a sample was removed through a 0.22‐*μ*m sterile filter, which removed the cells, and 10 *μ*L of the resultant supernatant was tested for the presence of formate by HPLC (Fig. 3A). Following 5 h incubation, a final concentration of 5.1 ± 1.2 mmol/L formate was calculated to be present in the cell suspension. Incubation of the RT1 strain in the absence of either H_2_ or CO_2_/H_2_ mix did not result in formate production (Fig. 3A). In addition, a control strain (RT2), which was genetically identical to RT1 but further deleted for the *hycABCDEFGHI* operon encoding the majority of FHL (Fig. 2), could not produce formate from CO_2_ and H_2_ under the same conditions (Fig. 3A) and contained only a trace of formate (0.6 ± 0.4 mmol/L final concentration in the reaction vessel), most likely as carry over from the initial growth phase. Lactate was always observed as a carryover even after extensive washing of the cells (Fig. 3A) and served as a useful internal control. The HPLC traces in Fig. 3A have some other interesting features. First, the small H_2_‐dependent (but CO_2_‐independent) formate peak is probably most likely due to the presence of internally produced CO_2_ by the cells, especially as the cells retain some viability and are actively excreting lactate (Fig. 3A). More intriguing is the presence of minor signals at 21 and 22 min retention time. Collecting the 22 min fraction and analysis by mass spectrometry gave no clear results. Sample compounds (oxaloacetate, fumarate, acetate, and various amino acids) all showed different retention times to this and can therefore be discounted. Thus, the definitive nature of the substance eluting at 22 min remains to be determined.

Next, CO_2_/H_2_‐dependent formate production by the RT1 strain was followed over time in order to gain insight into the reaction rate (Fig. 3B). At the beginning of the time course the RT1 strain showed a trace background level of formate (0.7 mmol/L) in the supernatant, which increased in a linear fashion over 3.5 h to reach 5.1 mmol/L (final concentration in the 3 mL aqueous phase of the reaction vessel), which is equivalent to an initial rate of 4 nmoles of formate produced *per* min *per* mg protein. The reaction slowed and reached a steady state after 10.5 h with a final concentration of 8.7 mmol/L formate in the reaction vessel (Fig. 3B). The production of formate is detectible but slow, and thus performing an HPLC‐based time course for many different experimental conditions was deemed not practical. Instead, a single‐point assay was chosen for further formate production experiments, where the concentration of formate in the aqueous phase would be determined after 5 h incubation.

Using the single‐point assay, the pH dependence of formate production from CO_2_/H_2_ was assessed (Fig. 3C). The reaction was found to be strongly dependent on the final pH of the buffer (Fig. 3C). Buffer systems with potassium phosphate for pH 5.5–7.4, MOPS buffer for pH 6.8–7.7, and boric acid buffer for pH 7.8–8.7 were tested. Due to the amount of CO_2_ that can dissolve in the aqueous phase, and thus potentially acidify the system, all pHs were remeasured after addition of CO_2_ and those values plotted against formate production (Fig. 3C). These experiments clearly showed that the amount of formate produced over the 5 h timescale peaked at a maximal level of production under pH 7.4 conditions (Fig. 3C).

The single‐point formate assay, together with further molecular genetics, was used to further dissect the formate production from CO_2_ and H_2_ activity exhibited by *E. coli*. Interestingly, the inactivation of the respiratory formate dehydrogenases (Δ*fdhE*) and the PFL enzymes (Δ*pflA*) in strain RT1 apparently had little effect on the amount of formate produced (Table 3). Under these anaerobic assay conditions in nutrient‐depleted buffer, the parent strain CP734 (Δ*hyaB*, Δ*hybC*) generated identical amounts of formate as the RT1 strain (Table 3). More importantly, the single‐point assay, together with further mutagenesis, was able to establish that formate production from CO_2_ and H_2_ was catalyzed specifically by FHL (Table 3). The RT1 stain was further modified to incorporate a complete deletion of the *hycABCDEFGHI* operon, encoding Hyd‐3, to give strain RT2 (Table 3). The new RT2 strain was unable to generate formate from H_2_/CO_2_ (Table 3).

**Table 3:** *In trans* complementation of a Δ*hycC* strain with *hycC* mutant genes

Strain + Complementing plasmid	Formate production (% of parental)^a^	H_2_ production (% of parental)^b^
JW2693 (Δ*hycC*)		
+pKS‐*hycCD* ^+^	89 ± 10	93 ± 3
+HycC (L208K)	129 ± 18	127 ± 47
+HycC (H215A)	139 ± 6	132 ± 46
+HycC (H332A)	118 ± 16	106 ± 33
+HycC (D354A)	99 ± 5	55 ± 18
+HycC (E391A)	86 ± 38	55 ± 19

a Activity was calculated based on a single‐point assay following incubation of equal amounts of pregrown cells washed and suspended in 3‐mL 20 mmol/L MOPS (pH 7.4) in sealed Hungate tubes containing a H_2_ and CO_2_ atmosphere. A value of 100% corresponds to 5.1 mmol/L formate (final concentration), which is the amount produced by an FHL^+^ parental strain following 5 h incubation. The ± values indicate the percentage of the standard deviation from the respective value (*n* > 3).

b Reaction rates were calculated using a continuous assay of pregrown, washed, live cells in a H_2_‐sensing electrode. The reaction was started by the addition of excess formate and an initial rate of the parental strain CP734 was 37.8 ± 7.6 nmol H_2_ produced min^−1^ mg^−1^, and this value was used to correspond to 100% activity. The ± values indicate the percentage of the standard deviation from the respective value (*n* > 3).

Taken together, these data clearly demonstrate that FHL is reversible in the intact cell and that CO_2_ initially applied to the gas phase can be reduced to formate in a H_2_‐dependent manner specifically by FHL. The reaction is nonphysiological, as *E. coli* would not normally express FHL at pH 7.4 (the optimum for the reaction) and low formate concentrations, and *E. coli* would not normally use formate as a carbon source. The reverse reaction is also relatively slow (4 nmol formate min^−1^ mg^−1^). To compare this reaction rate to the forward FHL reaction, the identical strains were assayed for formate‐dependent hydrogen production (Table 3). In this case, rates of 38 nmol H_2_ min^−1^ mg^−1^ were calculated, except for the RT2 control strain (Table 3). Thus, the forward reaction is an order of magnitude faster than the reverse reaction in intact cells.

### H_2_‐dependent formate generation from CO_2_ in vitro by purified FHL

**Figure 4:**
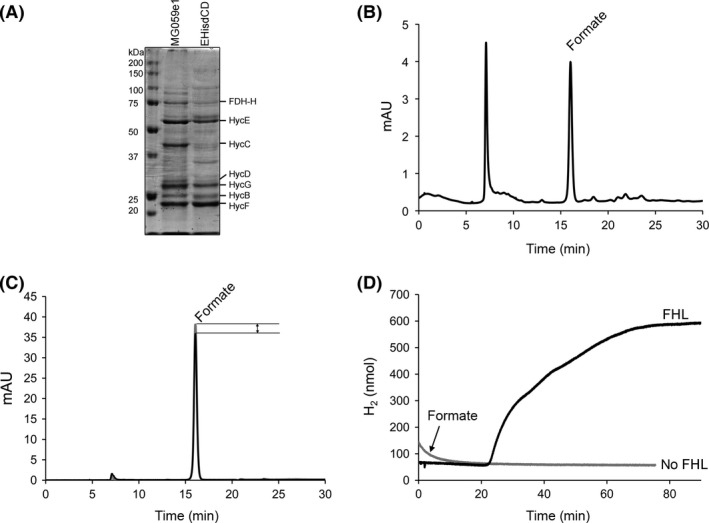
Reactions of purified formate hydrogenlyase (FHL‐) complex in vitro. (A) Purified protein (30 *μ*g) from strains MG059e1 (FHL with internal His‐tag on HycE) and EHisdCD (same as MG059e1, but with deletion of *hycCD*) was applied to a 10% (w/v acrylamide) SDS‐PAGE and separated. The molecular mass of the ladder is given on the left‐hand side (PageRuler Plus, Fisher Scientific). (B) A representative high‐performance liquid chromatography (HPLC) profile of formate production by purified FHL complex (370 *μ*g) after incubation under CO_2_ and H_2_ atmosphere for 5 h at 37°C. An aliquot of 10 *μ*L was applied directly to an Aminex HPX 87H column at 50°C and separated compounds detected at A_210 nm_. The formate peak elutes at 16.2 min, whereas the injection peak can be seen at 7 min. (C) Representative HPLC analysis of formate content before (gray, 15.74 mmol/L from a stock solution) and after incubation with 370‐*μ*g purified FHL complex (approx. 1.2 nmol) for 5 h at 37°C (black trace, 14.90 ± 0.07 mmol/L, *n* = 5, *P* < 0.01 using a paired *t*‐test). (D) A representative assay of the forward reaction using detergent‐solubilized, purified FHL (370 *μ*g) in a hydrogen‐sensing Clark‐type electrode. The trace (black) shows the reaction when initiated through the addition of 15 mmol/L (final concentration) formate. Strict anaerobiosis is required for this assay and this reaction was therefore carried out in the presence of an enzymatic oxygen scavenger system with D‐glucose, glucose oxidase, and catalase as described (Sargent et al. 1999). The lag time before the reaction commences is considered to be due to the time taken to reduce residual O_2_ in the reaction vessel to noninhibitory levels. The same oxygen‐scavenging components were added in the absence of FHL complex as a control (gray trace).

Recently, a strain and protocol has been reported that allows the intact isolation of *E. coli* FHL *via* an internal affinity tag located on the Hyd‐3 [NiFe]‐hydrogenase subunit (McDowall et al. 2014). The protocol involves rapid dispersal of cell membranes in a detergent cocktail prior to purification of the enzyme in dodecyl maltoside–containing buffer (McDowall et al. 2014). In order to test the ability of isolated FHL to perform CO_2_/H_2_‐dependent formate production, the enzyme was purified from *E. coli* strain MG059e1 (*hycE* ^His^) by immobilized metal‐affinity chromatography (IMAC) as previously described (McDowall et al. 2014). SDS‐PAGE analysis established that all seven subunits were present in the preparation (Fig. 4A). The Hyd‐3 activity was assayed (H_2_‐dependent benzyl viologen reduction) for this preparation and found to be 2.44 ± 0.62 U mg^−1^. In addition, formate‐dependent reduction in benzyl viologen activity (specific for the FDH‐H component of FHL) was assayed and recorded as 0.82 ± 0.04 U mg^−1^. These data give confidence that the as‐isolated FHL is enzymatically active. Next, the isolated FHL (370 *μ*g) was incubated in an atmosphere of CO_2_ and H_2_ before single‐point formate production was determined by HPLC. Here, the enzyme purified in detergent was able to produce 1.55 ± 0.01 mmol/L formate (final concentration) after 5 h incubation at 37°C. This corresponds to 3.25 *μ*mol formate produced in the reaction vessel (Fig. 4B). Therefore, from the initial 471 *μ*mol (39 mmol/L initial concentration) of CO_2_ added, only around 0.7% was converted over this time course.

For comparison, the relative rate of the formate‐dependent H_2_ production ‘forward’ reaction was monitored for the purified protein in vitro using discontinuous HPLC and continuous H_2_‐sensing electrode assays. Isolated enzyme (370 *μ*g) was incubated with formate and the concentration of the substrate was assessed by HPLC. The HPLC experiment showed that after 5 h under N_2_ atmosphere, the formate concentration decreased from 15.74 mmol/L to 14.90 ± 0.07 mmol/L (final concentration) in the reaction vessel (Fig. 4C). A more informative assay involves formate‐dependent H_2_ evolution directly in a H_2_‐detecting electrode cell (Fig. 4D). Here, detergent‐solubilized and purified FHL was incubated in solution in an anaerobic electrode chamber. The reaction was initiated by the addition of formate (15 mmol/L initial concentration) and an initial velocity of 103‐nmol H_2_ produced *per* min *per* mg enzyme was recorded (Fig. 4D). Assuming an intact molecular mass of 306 kDa for FHL (McDowall et al. 2015), the forward reaction turnover number can be estimated at 32 min^−1^.

These experiments corroborate results from the initial in vivo analysis (Fig. 2A) and show that isolated FHL is also reversible in vitro.

### The role of the membrane in FHL activity: effect of chemical uncouplers on FHL activity

The data presented here, and that previously reported (McDowall et al. 2014), clearly demonstrate that the FHL complex does not strictly rely on membrane attachment for activity. This raises questions on the involvement of the transmembrane electrochemical gradient and the function of the membrane‐embedded subunits themselves. However, the development here of assays for both the FHL forward and reverse reactions allows these questions to be addressed. In essence, if one direction of the FHL reaction was tightly coupled to proton or ion translocation in vivo then it follows that the opposite reaction direction should be dependent upon a transmembrane electrochemical gradient.

To begin to test this, the *E. coli* RT1 strain (Δ*fdhE,* Δ*hyaB*, Δ*hybC*, and Δ*pflA*) was employed to assay formate production from CO_2_ and H_2_ under different conditions (Table 4). Specifically, the effect of protonophores/ionophores on formate generation from CO_2_ and H_2_ was monitored by HPLC (Table 4). To do this, a single end‐point assay was used where formate production was determined after 5 h incubation. Such an assay is not appropriate for determining reaction rates *per se*, but instead gives a clear indication of whether FHL is active under the chosen conditions. The addition of CCCP (protonophore), nigericin (K^+^/H^+^ exchanger), valinomycin (K^+^ ionophore), gramicidin (K^+^ ionophore), or monensin and EIPA (both Na^+^/H^+^ exchangers) at levels found to be effective in previous studies was found to have no effect on the final amount of formate produced in this single‐point assay (Table 4). Also, the combination of EIPA with high external sodium ions, or valinomycin with high external potassium ions, did not increase the formate production by more than 12% and 21%, respectively (Table 4). The addition of 2,4‐dinitrophenol (a protonophore) reduced formate production to around 72% of the untreated level (Table 4). Thus, it can be concluded that the transmembrane electrochemical gradient is not strictly required to drive the FHL ‘reverse’ reaction. Likewise, there appears to be no obvious thermodynamic backpressure exerted on the FHL ‘reverse’ reaction by the transmembrane electrochemical gradient, as no significant increase in activity in the presence of uncouplers was observed.

**Table 4:** *In trans* complementation of a Δ*hycD* strain with *hycD* mutant genes

Strain + Complementing plasmid	Formate production (% of parental)^a^	H_2_ production (% of parental)^b^
JW2692 (Δ*hycD*)		
+pKS‐*hycCD* ^+^	103 ± 1	83 ± 20
+HycD (E138A)	26 ± 2	2 ± 2
+HycD (E189A)	87 ± 1	85 ± 17
+HycD (E199A)	133 ± 11	76 ± 5
+HycD (E201A)	82 ± 12	86 ± 12
+HycD (E203A)	18 ± 11	5 ± 7
+HycD (E199/201/203A)	27 ± 3	1 ± 1

a Activity was calculated based on a single‐point assay following incubation of equal amounts of pregrown cells washed and suspended in 3‐mL 20 mmol/L MOPS (pH 7.4) in sealed Hungate tubes containing a H_2_ and CO_2_ atmosphere. A value of 100% corresponds to 5.1 mmol/L formate (final concentration), which is the amount produced by an FHL^+^ parental strain following 5 h incubation. The ± values indicate the percentage of the standard deviation from the respective value (*n* > 3).

b Reaction rates were calculated using a continuous assay of pregrown, washed, live cells in a H_2_‐sensing electrode. The reaction was started by the addition of excess formate and an initial rate of the parental strain CP734 was 37.8 ± 7.6 nmol H_2_ produced min^−1^ mg^−1^, and this value was used to correspond to 100% activity. The ± values indicate the percentage of the standard deviation from the respective value (*n* > 3).

Next, the *E. coli* CP734 strain (Δ*hyaB* and Δ*hybC*) was used to assay formate‐dependent H_2_ evolution (the FHL ‘forward’ reaction) in a H_2_‐sensing electrode (Table 4). This is a continuous assay where reaction rates can be determined. The CP734 strain cannot generate H_2_ by reverse electron transport through Hyd‐2, as recently reported (Pinske et al. 2015), as this isoenzyme has been genetically inactivated. The CP734 (Δ*hyaB* and Δ*hybC*) strain was cultured under fermentative conditions, harvested and washed, before whole cells were assayed for H_2_ production activity under anaerobic conditions. In the presence of most chemical uncouplers tested, there was no change to the rates of H_2_ production by the FHL complex (Table 4); however, the addition of CCCP (a protonophore) reduced hydrogen production rates to around 58% of the native level (Table 4). Note that all experiments were also carried out in the presence of 1 mmol/L EDTA to ensure adequate access of the compounds to the cytoplasmic membrane. However, the detected FHL activities observed were identical to those measured in the absence of EDTA (data not shown). In must be concluded that the ‘forward’ reaction is not dependent on the transmembrane electrochemical gradient, as its collapse with chemical uncouplers has no obvious effect on H_2_ production rates from formate.

Finally, strains RT1 (Δ*fdhE*, Δ*hyaB*, Δ*hybC*, and Δ*pflA*) and CP734 (Δ*hyaB* and Δ*hybC*) were treated with DCCD, an aspartate cross‐linker that affects F_1_F_o_ ATP synthase activity; however, no effect on FHL activity was observed in these experiments (Table 4).

### The role of the membrane subunits in FHL activity: site‐directed mutagenesis of HycC

FHL is anchored to the inner membrane by a transmembrane complex of two proteins: HycC and HycD. An *E. coli* strain (EhisdCD) was constructed that carried in‐frame, unmarked deletions of the *hycCD* genes in the His‐tagged HycE background. Western immunoblot characterization of strain EHisdCD showed that there is no polar effect on HycG production, which is encoded downstream of the Δ*hycCD* allele (Fig. S1). The EhisdCD (Δ*hycCD*) double‐deletion strain was then assayed for in vivo FHL activity and it was observed that both formate‐dependent H_2_ production and CO_2_/H_2_‐dependent formate production were abolished in the mutant strain (Table 3). Similarly, *E. coli* strains carrying single deletion mutations in either *hycC* or *hycD* were also essentially devoid of FHL activity (Table 3).

The HycC protein is evolutionarily related to the NuoL/NuoM/NuoN proteins from the respiratory Complex I (Efremov and Sazanov 2012). Although the proton translocation mechanism of Complex I is not yet fully understood, two essential lysine side chains (K234 and K265), and one essential glutamate residue (E144), have been previously identified in *E. coli* NuoM that when substituted abolished quinone reductase activity by Complex I (Euro et al. 2008) and reduced or abolished proton‐pumping activity (Efremov and Sazanov 2011). *E. coli* HycC contains equivalents of these essential NuoM residues, namely HycC E135 (equivalent to NuoM E144) and K239 (equivalent to NuoM K265), whereas the corresponding residue to NuoM K234 is a leucine in HycC (L208). Initially, the CP734 (Δ*hyaB* and Δ*hybC*) strain was further modified by the separate introduction of alleles‐encoding HycC E135A and K239A variants (Table 3) onto the native chromosomal locus resulting in strains CPCE135A and CPCK239A, respectively. Following pregrowth, harvesting, and washing, the CPCE135A and CPCK239A strains were found to be able to perform CO_2_/H_2_‐dependent production of formate, using the single end‐point assay, to a similar level observed in the parent strain (Table 3). Also, the CPCE135A strain exhibited formate‐dependent H_2_ production activity of a similar rate of that measured for the parent strain (Table 3). Using the homologous recombination method chosen here to prepare genetically modified strains, it proved impossible to select a HycC L208K variant strain. As a compromise, the *hycC* L208K allele was prepared on a plasmid and used to complement a Δ*hycC* strain *in trans*. The HycC L208K variant (strain JW2693 with pKS‐hycC_L208K_D) function for the FHL ‘reverse’ reaction (formate production) was found to be slightly elevated compared to RT1 and the parent strain complemented with native *hycC* (Table 5).

**Table 5:** Strains used and constructed in this study

Strain	Genotype	Reference
MG059e1	As MG1655, *hycE* ^His^	McDowall et al. (2014)
EHisdCD	As MG059e1, Δ*hycCD*	This study
MC4100	F^−^, *araD139*, Δ(*argF‐lac*)*U169, ptsF25, deoC1, relA1, flbB5301, rspL150*	Casadaban (1976)
CP734	As MC4100, Δ*hyaB*, Δ*hybC*	Pinske et al. 2011b
CP971	As MC4100, Δ*hycA‐I*::*cat*	Pinske et al. (2011a)
JW2693	As BW25113, Δ*hycC*::*kan*	Baba et al. (2006)
JW2692	As BW25113, Δ*hycD*::*kan*	Baba et al. (2006)
RT1	As MC4100, Δ*hyaB,* Δ*hybC,* Δ*fdhE,* Δ*pflA*	This study
RT2	As MC4100, Δ*hyaB,* Δ*hybC,* Δ*fdhE,* Δ*pflA,* Δ*hycA‐I*::*kan*	This study
CPCT83A	As CP734, HycC_T83A_	This study
CPCE135A	As CP734, HycC_E135A_	This study
CPCH222A	As CP734, HycC_H222A_	This study
CPCK239A	As CP734, HycC_K239A_	This study
CPCE281A	As CP734, HycC_E281A_	This study
CPCT292A	As CP734, HycC_T292A_	This study
CPCE294A	As CP734, HycC_E294A_	This study
CPCN295A	As CP734, HycC_N295A_	This study
CPCH328A	As CP734, HycC_H328A_	This study
CPCK336A	As CP734, HycC_K336A_	This study
CPCN386A	As CP734, HycC_N386A_	This study
CPCF388A	As CP734, HycC_F388A_	This study

**Figure 5:**
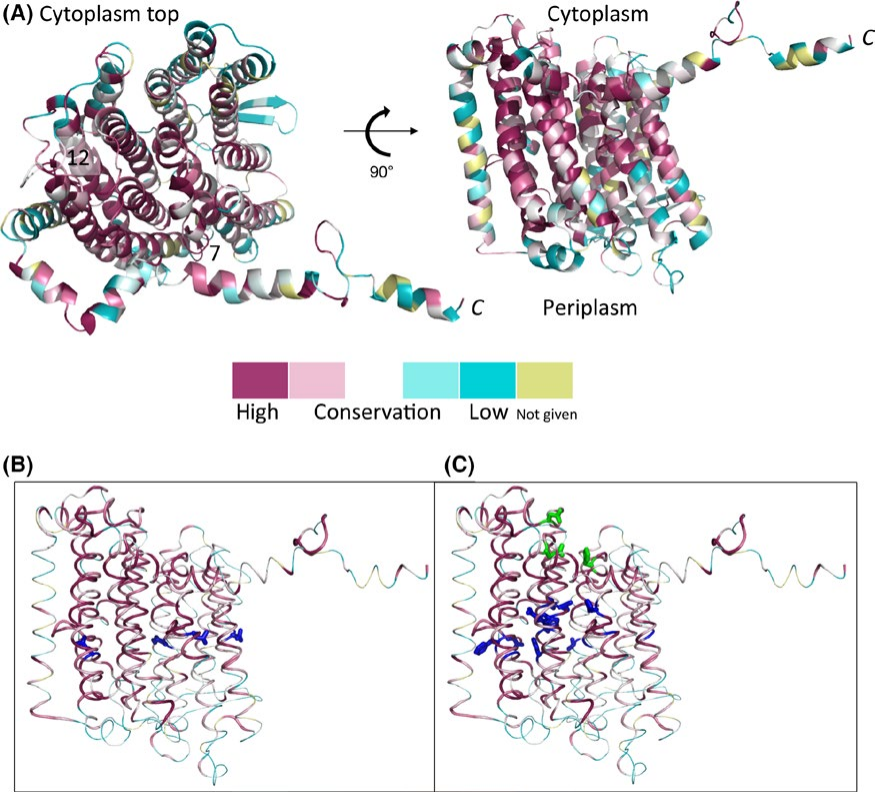
The predicted structure of HycC. (A) A cartoon representation of the Phyre^2^‐predicted HycC fold as seen from the cytoplasmic side or in the membrane plane, as indicated. The long‐extended *C*‐terminal helix is labeled. The amino acids are colored according to their ConSurf conservation scores with highest conservation being *red*. (B) The view within the membrane plane with amino acids E135, L208, K239, and E391 shown as *blue sticks*. (C) The amino acids H222, E281, and D354 in *green sticks*, whereas T83, H215, T292, E294, N295, H328, H332, K336, N386, and F388 are shown as *blue sticks*.

To explore further possible regions of HycC with functional importance, conservation scores for HycC amino acid residues were determined using ConSurf (Ashkenazy et al. 2010) based on sequence alignments among the *E. coli* NuoM, HyfB, and HycC proteins. Regions of conservation were plotted on a HycC structure model generated by the Phyre^2^ server (Kelley and Sternberg 2009) and from those a belt of conserved charged or polar residues predicted to reside within the lipid bilayer was identified (Fig. 5). This analysis identified HycC residues T83, H215, T292, E294, N295, H328, H332, K336, N386, and F388 and each was targeted for substitution by alanine. In addition, conserved polar residues predicted to lie on the cytoplasmic face of the HycC protein were identified, resulting in H222, E281, and D354 being chosen for substitution with alanine.

In total, 10 further derivatives of the CP734 (Δ*hyaB* and Δ*hybC*) strain were carefully constructed carrying unmarked alleles expressed at the native *hyc* locus on the chromosome. These new strains produced HycC variants T83A, H222A, E281A, T292A, E294A, N295A, H328A, K336A, N386A, and F388A. Of these 10 new strains, none was found to be impaired for CO_2_/H_2_‐dependent formate production using the single end‐point assay (Table 3). Similarly, the strains exhibited formate‐dependent H_2_ production rates similar to the parental strain CP734 (Table 3).

Of the remaining HycC residues targeted for substitution by alanine, the alleles‐encoding HycC H215A, H332A, D354A, and E391A were characterized by *in trans* complementation of a Δ*hycC* strain (Table 5). All were active for CO_2_/H_2_‐dependent formate production as assessed by the single end‐point assay, yielding final formate amounts between 86 and 139% of that generated by the parental strain CP734 (Table 5). However, analysis of formate‐dependent H_2_ production rates (the forward reaction) showed that D354A or E391A substitutions in HycC reduced H_2_ production to around 55% (Table 5).

### The role of the membrane subunits in FHL activity: site‐directed mutagenesis of HycD

The FHL membrane subunit HycD shares homology with NuoH from Complex I. This subunit has a remarkable amount of conserved glutamate residues that, in Complex I, have been proposed to transmit the change in charge during the reaction from the ‘N2’ [Fe‐S]‐cluster and quinone‐binding site to the membrane subunits (Efremov and Sazanov 2012). In *E. coli* Complex I, NuoH E157 and V206 were previously reported to be involved in energy coupling (Hirst 2013). Here, the effect of substituting the equivalent conserved residues E138, E189 (predicted to lie within the lipid bilayer), and the motif E199/201/203 (predicted to be in an exposed, cytoplasmic loop), in HycD, was investigated. Note that sequence analysis suggests that *E. coli* HycD E138 and E189 adopt the positions of residues E157 and V206 in *E. coli* NuoH, respectively. The data show that the HycD E189A, E199A, and E201A variants retained activity for both FHL ‘forward’ and ‘reverse’ reactions, whereas the E138A, E203A, and the E199/201/203A triple exchange had reduced activities for the both the ‘forward’ and ‘reverse’ reactions (Table 6).

**Table 6:** Plasmids used in this study

Plasmid	Genotype	Reference
pKS‐hycCD	pBluescript KS+; *hycCD* ^+^ including 500 bp flanking regions; Amp^R^	This study
pKS‐hycB:E	pBluescript KS+; *hycB*::*E* Δ*hycCD*; Amp^R^	This study
pMAK705	Cm^R^	Hamilton et al. (1989)
pMAK‐hycB::E	pMAK705; *hycB*::*E* Δ*hycCD*; Cm^R^	This study
pKS‐hycC_L208K_D	pKS‐hycCD, HycC (L208K)	This study
pKS‐hycC_H215A_D	pKS‐hycCD, HycC (H215A)	This study
pKS‐hycC_H332A_D	pKS‐hycCD, HycC (H332A)	This study
pKS‐hycC_D354A_D	pKS‐hycCD, HycC (D354A)	This study
pKS‐hycC_E391A_D	pKS‐hycCD, HycC (E391A)	This study
pKS‐hycCD_E138A_	pKS‐hycCD, HycD (E138A)	This study
pKS‐hycCD_E189A_	pKS‐hycCD, HycD (E189A)	This study
pKS‐hycCD_E199A_	pKS‐hycCD, HycD (E199A)	This study
pKS‐hycCD_E201A_	pKS‐hycCD, HycD (E201A)	This study
pKS‐hycCD_E203A_	pKS‐hycCD, HycD (E203A)	This study
pKS‐hycCD_E199/201/203A_	pKS‐hycCD, HycD (E199/201/203A)	This study

### A variant of FHL lacking integral membrane subunits can be purified, but has low activity

As deletion of the *hycC* and *hycD* genes abolishes FHL function in intact whole cells (Table 3), it was considered a possibility that the remaining FHL subunits are not synthesized or assembled in the mutant strain. To address this, the *E. coli* EhisdCD strain (Δ*hycC*, Δ*hycD*, and *hycE* ^His^) was cultured under fermentative conditions and the variant enzyme purified by IMAC. Subsequent SDS‐PAGE analysis of the isolated protein showed that the entire five‐subunit soluble domain of FHL could be copurified with the single affinity tag on HycE (Fig. 4A). In this case, however, the BV redox dye‐linked Hyd‐3 activity was assayed as 0.12 U mg^−1^, which represents only 5% of activity normally measured for the entire enzyme complex, and the BV redox dye‐linked FDH‐H activity was recorded as 0.40 ± 0.09 U mg^−1^, which is 49% of the activity normally associated with the native complex. Thus, in the complete absence of the membrane domain, the FHL cytoplasmic domain is apparently assembled but clearly compromised in terms of its enzymatic activity.

Similarly, purification of HycE^His^ after transfer of the allele encoding the HycD E199/201/203A triple exchange to the MG059e1 chromosome allowed purification of an assembled five‐subunit soluble domain that displays very low enzymatic activity. The HycD E199/201/203A strain hence phenocopies the Δ*hycCD* deletion strain (data not shown).

## Discussion

### Formate hydrogenlyase can be made to perform the reverse reaction

The physiological role of FHL is to oxidize formate with the concomitant production of molecular hydrogen. Indeed, gene regulation and enzyme assembly have evolved to such an extent that the FHL enzyme is only synthesized under relatively high formate concentration and relatively low pH (Rossmann et al. 1991; Sawers 1994; Suppmann and Sawers 1994). In the main, therefore, the ‘forward’ reaction catalyzed by FHL (H_2_ production using formate as an electron donor) has been the focus of research in this area. However, understanding and harnessing the ‘reverse’ FHL reaction (H_2_‐driven reduction in CO_2_ to formate) are potentially of great interest in terms of carbon capture and the generation of sustainable chemical feedstocks for industry. As Van't Hoff stated in 1884, every chemical reaction should be reversible, and this of course also applies to biological systems as enzymes do not shift the chemical equilibrium, but instead lower the activation energy. It was therefore important to demonstrate experimentally that FHL could operate in ‘reverse’ given the correct conditions.

The data presented in this work clearly show that *E. coli* FHL can reduce CO_2_ to formate using H_2_ as electron donor. The discontinuous assay employed here suggests the reaction is slow (4 nmol formate produced min^−1^ mg^−1^) compared to the forward reaction, which was recorded here as 38 nmol H_2_ produced min^−1^ mg^−1^ but has been reported as high as 500 nmol H_2_ produced min^−1^ mg^−1^ (Sawers et al. 1985). A turnover number for the forward reaction was estimated in this work as 32 min^−1^, which is in agreement with previous work using a different assay (McDowall et al. 2014). It is perhaps not surprising that the reverse reaction is at least an order of magnitude slower than the forward reaction as this direction of electron flow is never normally attempted by FHL – the reaction is nonphysiological and *E. coli* would not normally attempt to fix CO_2_ in this way. In these experiments, cells have been pregrown to induce FHL production and then placed under unusual conditions (high CO_2_/H_2_ and alkaline pH) that would favor the reverse reaction. It should also be considered that the, currently unknown, individual redox potentials of the multiple metallocofactors in FHL are biased toward performing the forward reaction. This, together with other enzyme‐gating mechanisms, may slow the reverse reaction significantly. It is conceivable, therefore, that future synthetic biology approaches could be used to remove the natural gene expression restrictions from FHL biosynthesis, modify the cofactor coordination chemistry, and so engineer a strain that is primed to perform the ‘reverse’ reaction.

### No evidence for proton translocation by the *E. coli* FHL complex

The evolutionary link between FHL and respiratory Complex I has been a source of fascination and experimentation since the primary sequence of FHL components was first revealed (Böhm et al. 1990). In recent years great strides forward have been made in understanding the structure and function of Complex I. It has been suggested for Complex I that in a two‐state stabilization charge mechanism, where negatively charged quinone stabilization drives conformational changes to transduce energy from the soluble domain to the membrane domain in order to pump protons (Brandt 2011; Zickermann et al. 2015). However, no involvement of quinone, or a quinone‐binding site, has been shown for FHL, although sequence analysis has hinted at the possibility (Weiss et al. 1991). In Complex I, the quinone‐binding site was revealed to be located close to the terminal [Fe‐S]‐cluster named N2 in Complex I, which corresponds to the proximal [Fe‐S] cluster of the Hyd‐3 small‐subunit HycG that is predicted to be intimately linked with the [NiFe] active site in the large‐subunit HycE. Therefore, the [NiFe] catalytic center in HycE occupies the space where quinone binding takes place in Complex I. Thus, in this case the proposed charge transfer to the membrane domain would result from the quenching of two positively charged protons by reducing them to dihydrogen in HycE close to HycD. The residues proposed to be required for charge transfer from Complex I subunit NuoH are equally conserved in HycD, but single substitutions generated in this work had very little effect on activity, or potentially the loss of a single charge can be compensated for within the wider structure of the FHL membrane domain.

Hyd‐3 was the first described example of ‘group 4’ [NiFe] hydrogenases also named Ech (energy‐converting Hydrogenase) (Wu and Mandrand‐Berthelot 1993; Vignais et al. 2001). This group also comprises the Ech‐Hyd from *Methanosarcina barkeri* and the membrane‐bound Hyd from *Pyrococcus furiosus* where energy conversion as a build‐up of a proton/Na^+^ gradient was shown using inverted‐membrane vesicles (Sapra et al. 2003; Hedderich and Forzi 2005). Several studies have attempted to demonstrate proton pumping by *E. coli* FHL (Bagramyan and Martirosov 1989; Trchounian et al. 2000; Bagramyan et al. 2002; Hakobyan et al. 2005). Early studies correlated the time for H^+^/K^+^ pumping activity with FHL activity (Bagramyan and Martirosov 1989) and discovered that the redox potential, which was correlated or associated with H_2_ production, was sensitive to the ATPase‐inhibitor DCCD (*N,N*’‐ dicyclohexylmethanediimine). Also, mutants without functional F_1_F_o_‐ATPase were apparently affected in H_2_ production by Hyd‐4 (a subunit of the poorly understood alternative ‘FHL‐2’ system), but had no influence on Hyd‐3 (Bagramyan et al. 2002). Thus, a model was proposed where the ATP‐dependent potassium‐transporter TrkA and the F_1_F_o_‐ATPase could form a supercomplex with FHL‐2 and possibly with FHL itself in order to couple H_2_ production and H^+^/K^+^ exchange to a proton gradient generation (for reviews see Bagramyan and Trchounian 2003; Trchounian and Sawers 2014). In addition, the oxidation of formate was correlated with a ratio of 1.3 protons translocated per molecule formate and was not detectable in a FHL^−^ strain (Hakobyan et al. 2005).

Complex I is reversible and has been shown to transfer electrons from succinate *via* quinol to NAD^+^ driven by Δp – classical reversed electron transport (reviewed in Brandt 2006; Hirst 2013). This ‘reverse’ reaction would be expected to be inhibited by uncouplers, and concomitantly the ‘forward’ reaction of NADH oxidation coupled to proton translocation is not inhibited by protonophores such as CCCP (Ripple et al. 2013). Given the development here of robust strains and assays for both directions of FHL, it was considered timely to revisit the bioenergetic requirements of *E. coli* FHL. The initial assumption made was that if FHL pumps protons or other ions across the cytoplasmic membrane during catalysis, then according to the concept of reversibility, the other direction would require an electrochemical gradient. Therefore, both directions of the FHL reaction were performed in the presence of protonophores and ionophores.

When the effect of protonophores CCCP and 2,4‐dinitrophenol was investigated, the formate‐dependent H_2_ production from whole cells was reduced to 42% of native levels with CCCP, but unaffected in the presence of 2,4‐dinitrophenol. Note that the amount of CCCP used here was found to be sufficient to block the pmf‐dependent H_2_ production activity of *E. coli* Hyd‐2 in intact cells (Pinske et al. 2015). The discrepancy between the CCCP and dinitro phenol (DNP) results was surprising as both compounds should have the same uncoupling effect on the membrane. It was also surprising that CCCP inhibits the ‘forward’ reaction only partially – and an IC_50_ value can be calculated at 100 *μ*mol/L (Fig. S2B). In agreement with this observation, the ‘reverse’ reaction is slightly elevated in the presence of CCCP (perhaps indicative of a relaxing of thermodynamic backpressure on the enzyme), but again 2,4‐dinitrophenol has no effect. Where CCCP effects on H_2_ production have been previously investigated, the redox potential *E*_*h*_ was monitored (rather than the presence of H_2_ directly) and correlated with H_2_ production leading to the observation that CCCP slowed the reduction in *E*_*h*_ to about −600 mV lower than normal, but not completely abolishing it (Bagramyan and Martirosov 1989). Similarly, proton‐pumping activity during formate oxidation was reported to be inhibited by CCCP (Hakobyan et al. 2005); however, in this case the experiment was not sufficiently genetically controlled, as the activity of the periplasmically located electrogenic formate dehydrogenases was not taken into account. Indeed, it was equally shown in the same study that CCCP had no effect on formate oxidation or H_2_ production (Hakobyan et al. 2005). It is possible that CCCP is having secondary side effects on the FHL activity recorded here. An often overlooked aspect of the FHL system is the need for formate transport across the inner membrane. The mechanism of gating of the FocA formate channel is not fully understood, but is thought to be sensitive to pH changes. Although formate transport is thought to be not directly dependent upon proton cotranslocation (Suppmann and Sawers 1994; Doberenz et al. 2014), it is possible the CCCP is having a nonspecific effect on channel activity that is leading to changes in substrate availability to the FHL enzyme.

When DCCD was added to the cells, which is a protein‐modifying reagent frequently used to inhibit the proton‐translocating ATPase, no alteration for FHL function was visible here. Similarly, other K^+^ (nigericin, valinomycin, and gramicidin) and Na^+^ (monensin and EIPA) ionophores had no effect on FHL‐dependent H_2_ production from formate. Taken altogether, these data suggest that FHL activity is not tightly coupled to proton translocation in *E. coli*. Indeed, studies on chemical [NiFe]‐hydrogenase models showed that formate‐dependent H_2_ production is possible without additional energy input (Nguyen et al. 2014) and final corroborating evidence comes from the recent introduction of an active proton‐pumping proteorhodopsin in *E. coli*, which had no influence on H_2_ production from FHL (Kuniyoshi et al. 2015).

### Mutagenesis of HycC

In this work a HycC tertiary structure prediction, using the Phyre^2^ server (Kelley and Sternberg 2009), was used as a model to design mutagenic experiments (Fig. 5). HycC is predicted to comprise at least 15 transmembrane helices and a long amphipathic helix (Fig. 4A). The model is missing 16 amino acids at the *C*‐terminus and leaves the possibility for another transmembrane helix. A recent study (Zhu and Vik 2015) contradicts the previously assumed role of the amphipathic helix in Complex I to confer conformational changes among the membrane subunits (Efremov et al. 2010) and supports the idea of this helix stabilizing the structure of the membrane domain (Brandt 2013). Other similarities between HycC and the NuoL/M/N proteins are the broken helices 7 and 12, perhaps indicative of proton‐gating mechanism requiring lysine or glutamate residues (Batista et al. 2013). However, one of the essential gating residues K234 in NuoM is not conserved in *E. coli* HycC, although the overall conservation within the wider HycC family is high (Marreiros et al. 2012).

Unlike experiments with Complex I, none of the 18 amino acid substitutions made in HycC in this work was found to have any effect on the FHL complex. It is arguable that the single amino acid residue exchanges in HycC might not be sufficient to interrupt a Grotthus proton translocation mechanism, although it was shown that single homologous residue exchanges in the Na^+^/H^+^ antiporter‐like subunits of Complex I are sufficient to stop proton translocation activity completely (Efremov and Sazanov 2011). It is more likely that these data corroborate the experiments with chemical uncouplers somewhat and highlight that many aspects of HycC fine structure are not essential for the forward or reverse reactions of *E. coli* FHL.

### FHL activity requires a membrane attachment step

FHL is undoubtedly a membrane‐bound enzyme, however, if membrane association is not fulfilling a bioenergetic role, as suggested here by the uncoupler experiments and HycC mutagenesis, then understanding the function, and the conservation, of the integral membrane domain becomes an interesting puzzle. In experiments reported here, it is clear both forward and reverse FHL reactions can be performed in vitro from purified and detergent‐solubilized enzyme showing that a membrane attachment is not necessary for FHL activity. Indeed, both directions of reaction were also detectable in crude extracts, that is, in a mixture of cytoplasm and membrane vesicles (Table 4). In such crude extracts, the generation of formate from CO_2_/H_2_ was 75% of the value recorded for intact RT1 (Δ*hyaB hybC fdhE pflA*) cells, and the H_2_ production was reduced to around 12% compared to whole CP734 (Δ*hyaB* Δ*hybC*) cells. The latter reduction in activity is presumably because, unlike for the HPLC experiments, the crude extracts were not degassed prior to application to the electrode and the in vitro FHL reaction is dependent on O_2_‐free conditions. Nevertheless, data reported here clearly show the membrane subunits are critical for in vivo FHL activity (Table 3), as genetic removal of either or both of HycCD results in almost complete absence of FHL activity in either direction. Note also that the two half reactions are still detectable with artificial redox dyes, but the values were very low in terms of Hyd‐3, in the absence of HycC or HycD and that an intact FHL soluble domain can be purified from the mutant cells. These data are in good agreement with previous experiments that showed that absence of either HycC or HycD did not reduce the formate dehydrogenase H activity, but abolished most of the total Hyd‐3 activity (Sauter et al. 1992).

These data were corroborated by site‐directed experiments conducted here on the HycD protein. In terms of the HycD subunit, amino acid exchanges of E138, predicted to be in a transmembrane domain, and E203, part of a cytoplasmic loop, led to obvious changes in FHL activity (Table 6). However, given both directions of the FHL reaction were affected by these substitutions, this may be due to the lack of FHL complex stability or problems with biosynthesis of the enzyme. Indeed, the three HycD residues E199, E201, and E203 are predicted to be located in a cytoplasmic loop at the interface with the FHL soluble domain, and the triple exchange mutant was found to behave in an identical manner to a Δ*hycCD* strain. Again, the HycD E199/201/203A strain displayed low levels of in vivo FHL activity, but the soluble domain was assembled but largely inactive with regards to the Hyd‐3 component. Thus, mutagenesis of *hycD* may destabilize the membrane domain leading to separation of the soluble and membrane domains.

Thus, production of the FHL soluble domain in the absence of a membrane domain leads to fully assembled protein, but which is largely inactive; whereas production of the soluble domain in the presence of a membrane domain leads to fully active enzyme that retains its activity even after membrane dispersal with detergents. Moreover, cytoplasmic analogs of FHL exist in other bacteria. For example, the hydrogen‐dependent CO_2_ reductase from *Acetobacterium woodii* comprises a molybdenum‐dependent formate dehydrogenase linked to an [FeFe]‐hydrogenase but operates freely in the cell cytoplasm (Schuchmann and Müller 2013). Taken altogether, it seems likely that membrane attachment of the *E. coli* FHL soluble domain is important for either maintaining activity of the soluble domain or is an important final step in the biosynthesis and activation of the enzyme.

### Concluding remarks

The data presented suggest that direct proton translocation through the membrane domain of FHL is not strictly coupled to the catalytic cycle of the FHL soluble domain. Instead, the membrane domain structure may be conserved as it is either required for final activation of the enzyme or because positioning of FHL at the membrane surface increases efficiency of formate oxidation after import. Future challenges will revolve around understanding the biochemical roles of the membrane subunits and establishing why the prominent nest of charged residues within the lipid bilayer has been strictly conserved. It may also be the case that conformational changes in the membrane domain are required to modulate electron transfer in the cytoplasmic domain, especially as mobile side chains located between cofactors are beginning to emerge as important players in the hydrogenase reaction mechanism (Bowman et al. 2014; Frielingsdorf et al. 2014) and the Hyd‐3 small and large subunits are predicted to contact HycD at the membrane.

The unambiguous demonstration that FHL can operate in the fixing of CO_2_ to formate both in vivo and in vitro is an important finding and could potentially outcompete cell‐based systems that rely on the addition of ionophores in order to have biotechnologically relevant formate generation (Schuchmann and Müller 2013). Unambiguous demonstration of the FHL reverse reaction also corroborates some hypotheses on the origins of life (Nitschke and Russell 2009) and it opens new doors for the engineering and harnessing of this enzyme for industrial applications.

## Conflict of Interest

The authors declare no conflicts of interest.

## Supporting information


**Figure S1.** Analysis of downstream polar effects.
**Figure S2.** Inhibition of H_2_ production through addition of protonophore CCCP (Carbonyl cyanide *m*‐chlorophenyl hydrazone).
**Table S1.** Oligonucleotides used in this work.

## References

[mbo3365-bib-0001] Andrews, S. C. , B. C. Berks , J. McClay , A. Ambler , M. A. Quail , P. Golby , et al. 1997 A 12‐cistron *Escherichia coli* operon (*hyf*) encoding a putative proton‐translocating formate hydrogenlyase system. Microbiology 143:3633–3647. 9387241 10.1099/00221287-143-11-3633

[mbo3365-bib-0002] Ashkenazy, H. , E. Erez , E. Martz , T. Pupko , and N. Ben‐Tal . 2010 ConSurf 2010: calculating evolutionary conservation in sequence and structure of proteins and nucleic acids. Nucleic Acids Res. 38:W529–W533. 20478830 10.1093/nar/gkq399 PMC2896094

[mbo3365-bib-0003] Baba, T. , T. Ara , M. Hasegawa , Y. Takai , Y. Okumura , M. Baba , et al. 2006 Construction of *Escherichia coli* K‐12 in‐frame, single‐gene knockout mutants: the Keio collection. Mol. Syst. Biol. 2:0008. 16738554 10.1038/msb4100050 PMC1681482

[mbo3365-bib-0004] Bagramyan, K. A. , and S. M. Martirosov . 1989 Formation of an ion transport supercomplex in *Escherichia coli*. An experimental model of direct transduction of energy. FEBS Lett. 246:149–152. 2468524 10.1016/0014-5793(89)80272-8

[mbo3365-bib-0005] Bagramyan, K. , and A. Trchounian . 2003 Structural and functional features of formate hydrogen lyase, an enzyme of mixed‐acid fermentation from *Escherichia coli* . Biochemistry (Mosc) 68:1159–1170. 14640957 10.1023/b:biry.0000009129.18714.a4

[mbo3365-bib-0006] Bagramyan, K. , N. Mnatsakanyan , A. Poladian , A. Vassilian , and A. Trchounian . 2002 The roles of hydrogenases 3 and 4, and the FoF_1_‐ATPase, in H_2_ production by *Escherichia coli* at alkaline and acidic pH. FEBS Lett. 516:172–178. 11959127 10.1016/s0014-5793(02)02555-3

[mbo3365-bib-0007] Bassegoda, A. , C. Madden , D. W. Wakerley , E. Reisner , and J. Hirst . 2014 Reversible interconversion of CO_2_ and formate by a molybdenum‐containing formate dehydrogenase. J. Am. Chem. Soc. 136:15473–15476. 25325406 10.1021/ja508647u

[mbo3365-bib-0008] Batista, A. P. , B. C. Marreiros , and M. M. Pereira . 2013 The antiporter‐like subunit constituent of the universal adaptor of Complex I, group 4 membrane‐bound [NiFe]‐hydrogenases and related complexes. Biol. Chem. 394:659–666. 23509215 10.1515/hsz-2012-0342

[mbo3365-bib-0009] Begg, Y. , J. Whyte , and B. Haddock . 1977 The identification of mutants of *Escherichia coli* deficient in formate dehydrogenase and nitrate reductase activities using dye indicator plates. FEMS Microbiol. Lett. 2:47–50.

[mbo3365-bib-0010] Bettenbrock, K. , H. Bai , M. Ederer , J. Green , K. J. Hellingwerf , M. Holcombe , et al. 2014 Towards a systems level understanding of the oxygen response of *Escherichia coli* . Adv. Microb. Physiol. 64:65–114. 24797925 10.1016/B978-0-12-800143-1.00002-6

[mbo3365-bib-0011] Böhm, R. , M. Sauter , and A. Böck . 1990 Nucleotide sequence and expression of an operon in *Escherichia coli* coding for formate hydrogenlyase components. Mol. Microbiol. 4:231–243. 2187144 10.1111/j.1365-2958.1990.tb00590.x

[mbo3365-bib-0012] Bowman, L. , L. Flanagan , P. K. Fyfe , A. Parkin , W. N. Hunter , and F. Sargent . 2014 How the structure of the large subunit controls function in an oxygen‐tolerant [NiFe]‐hydrogenase. Biochem. J. 458:449–458. 24428762 10.1042/BJ20131520 PMC3940037

[mbo3365-bib-0013] Brandt, U. 2006 Energy converting NADH:quinone oxidoreductase (complex I). Annu. Rev. Biochem. 75:69–92. 16756485 10.1146/annurev.biochem.75.103004.142539

[mbo3365-bib-0014] Brandt, U. 2011 A two‐state stabilization‐change mechanism for proton‐pumping complex I. Biochim. Biophys. Acta 1807:1364–1369. 21565159 10.1016/j.bbabio.2011.04.006

[mbo3365-bib-0015] Brandt, U. 2013 Inside view of a giant proton pump. Angew. Chem. Int. Ed. Engl. 52:7358–7360. 23703893 10.1002/anie.201303403

[mbo3365-bib-0016] Casadaban, M. J. 1976 Transposition and fusion of the lac genes to selected promoters in *Escherichia coli* using bacteriophage lambda and Mu. J. Mol. Biol. 104:541–555. 781293 10.1016/0022-2836(76)90119-4

[mbo3365-bib-0017] Deplanche, K. , I. Caldelari , I. P. Mikheenko , F. Sargent , and L. E. Macaskie . 2010 Involvement of hydrogenases in the formation of highly catalytic Pd(0) nanoparticles by bioreduction of Pd(II) using *Escherichia coli* mutant strains. Microbiology 156:2630–2640. 20542928 10.1099/mic.0.036681-0

[mbo3365-bib-0018] Doberenz, C. , M. Zorn , D. Falke , D. Nannemann , D. Hunger , L. Beyer , et al. 2014 Pyruvate formate‐lyase interacts directly with the formate channel FocA to regulate formate translocation. J. Mol. Biol. 426:2827–2839. 24887098 10.1016/j.jmb.2014.05.023 PMC5560055

[mbo3365-bib-0019] Efremov, R. G. , and L. A. Sazanov . 2011 Structure of the membrane domain of respiratory complex I. Nature 476:414–420. 21822288 10.1038/nature10330

[mbo3365-bib-0020] Efremov, R. G. , and L. A. Sazanov . 2012 The coupling mechanism of respiratory complex I ‐ A structural and evolutionary perspective. Biochim. Biophys. Acta 1817:1785–1795. 22386882 10.1016/j.bbabio.2012.02.015

[mbo3365-bib-0021] Efremov, R. G. , R. Baradaran , and L. A. Sazanov . 2010 The architecture of respiratory complex I. Nature 465:441–445. 20505720 10.1038/nature09066

[mbo3365-bib-0022] Euro, L. , G. Belevich , M. I. Verkhovsky , M. Wikström , and M. Verkhovskaya . 2008 Conserved lysine residues of the membrane subunit NuoM are involved in energy conversion by the proton‐pumping NADH:ubiquinone oxidoreductase (Complex I). Biochim. Biophys. Acta 1777:1166–1172. 18590697 10.1016/j.bbabio.2008.06.001

[mbo3365-bib-0023] Frielingsdorf, S. , J. Fritsch , A. Schmidt , M. Hammer , J. Löwenstein , E. Siebert , et al. 2014 Reversible [4Fe‐3S] cluster morphing in an O2‐tolerant [NiFe] hydrogenase. Nat. Chem. Biol. 10:378–385. 24705592 10.1038/nchembio.1500

[mbo3365-bib-0024] Guest, J. R. 1969 Biochemical and genetic studies with nitrate reductase C‐gene mutants of *Escherichia coli* . Mol. Gen. Genet. 105:285–297. 4904136 10.1007/BF00277583

[mbo3365-bib-0025] Hakobyan, M. , H. Sargsyan , and K. Bagramyan . 2005 Proton translocation coupled to formate oxidation in anaerobically grown fermenting *Escherichia coli* . Biophys. Chem. 115:55–61. 15848284 10.1016/j.bpc.2005.01.002

[mbo3365-bib-0026] Hamilton, C. M. , M. Aldea , B. K. Washburn , P. Babitzke , and S. R. Kushner . 1989 New method for generating deletions and gene replacements in *Escherichia coli* . J. Bacteriol. 171:4617–4622. 2548993 10.1128/jb.171.9.4617-4622.1989 PMC210259

[mbo3365-bib-0027] Hedderich, R. , and L. Forzi . 2005 Energy‐converting [NiFe] hydrogenases: more than just H_2_ activation. J. Mol. Microbiol. Biotechnol. 10:92–104. 16645307 10.1159/000091557

[mbo3365-bib-0028] Hirst, J. 2013 Mitochondrial complex I. Annu. Rev. Biochem. 82:551–575. 23527692 10.1146/annurev-biochem-070511-103700

[mbo3365-bib-0029] Kelley, L. A. , and M. J. E. Sternberg . 2009 Protein structure prediction on the web: a case study using the Phyre server. Nat. Protoc. 4:363–371. 19247286 10.1038/nprot.2009.2

[mbo3365-bib-0030] Khangulov, S. V. , V. N. Gladyshev , G. C. Dismukes , and T. C. Stadtman . 1998 Selenium‐containing formate dehydrogenase H from *Escherichia coli*: a molybdopterin enzyme that catalyzes formate oxidation without oxygen transfer. Biochemistry 37:3518–3528. 9521673 10.1021/bi972177k

[mbo3365-bib-0031] Kim, Y. J. , H. S. Lee , E. S. Kim , S. S. Bae , J. K. Lim , R. Matsumi , et al. 2010 Formate‐driven growth coupled with H_2_ production. Nature 467:352–355. 20844539 10.1038/nature09375

[mbo3365-bib-0032] Knappe, J. , F. A. Neugebauer , H. P. Blaschkowski , and M. Gänzler . 1984 Post‐translational activation introduces a free radical into pyruvate formate‐lyase. Proc. Natl Acad. Sci. USA 81:1332–1335. 6369325 10.1073/pnas.81.5.1332 PMC344827

[mbo3365-bib-0033] Krasna, A. 1984 Mutants of *Escherichia coli* with altered hydrogenase activity. J. Gen. Microbiol. 130:779–787. 6376698 10.1099/00221287-130-4-779

[mbo3365-bib-0034] Kuniyoshi, T. M. , A. Balan , A. C. G. Schenberg , D. Severino , and P. C. Hallenbeck . 2015 Heterologous expression of proteorhodopsin enhances H2 production in *Escherichia coli* when endogenous Hyd‐4 is overexpressed. J. Biotechnol. 206:52–57. 25913175 10.1016/j.jbiotec.2015.04.009

[mbo3365-bib-0035] Laemmli, U. 1970 Cleavage of structural proteins during the assembly of the head of bacteriophage T4. Nature 227:680–685. 5432063 10.1038/227680a0

[mbo3365-bib-0036] Lim, J. K. , F. Mayer , S. G. Kang , and V. Müller . 2014 Energy conservation by oxidation of formate to carbon dioxide and hydrogen via a sodium ion current in a hyperthermophilic archaeon. Proc. Natl Acad. Sci. USA 111:11497–11502. 25049407 10.1073/pnas.1407056111 PMC4128143

[mbo3365-bib-0037] Maeda, T. , V. Sanchez‐Torres , and T. Wood . 2007 *Escherichia coli* hydrogenase 3 is a reversible enzyme possessing hydrogen uptake and synthesis activities. Appl. Microbiol. Biotechnol. 76:1035–1042. 17668201 10.1007/s00253-007-1086-6

[mbo3365-bib-0038] Marreiros, B. C. , A. P. Batista , A. M. S. Duarte , and M. M. Pereira . 2012 A missing link between complex I and group 4 membrane‐bound [NiFe] hydrogenases. Biochim. Biophys. Acta 1827:198–209. 23000657 10.1016/j.bbabio.2012.09.012

[mbo3365-bib-0039] McDowall, J. S. , B. J. Murphy , M. Haumann , T. Palmer , F. A. Armstrong , and F. Sargent . 2014 Bacterial formate hydrogenlyase complex. Proc. Natl Acad. Sci. USA 111:E3948–E3956. 25157147 10.1073/pnas.1407927111 PMC4183296

[mbo3365-bib-0101] McDowall, J.S. , M.C. Hjersing , T. Palmer , and F. Sargent . 2015 Dissection and engineering of the *Escherichia coli* formate hydrogenlyase complex. FEBS Lett 589:3141–3147. 26358294 10.1016/j.febslet.2015.08.043

[mbo3365-bib-0040] Miller, J . 1972 Experiments in Molecular Genetics.

[mbo3365-bib-0041] Nguyen, N. T. , Y. Mori , T. Matsumoto , T. Yatabe , R. Kabe , H. Nakai , et al. 2014 A [NiFe] hydrogenase model that catalyses the release of hydrogen from formic acid. Chem. Commun. 50:13385–13387. 10.1039/c4cc05911e 25234420

[mbo3365-bib-0042] Nitschke, W. , and M. J. Russell . 2009 Hydrothermal focusing of chemical and chemiosmotic energy, supported by delivery of catalytic Fe, Ni, Mo/W Co, S and Se, forced life to emerge. J. Mol. Evol. 69:481–496. 19911220 10.1007/s00239-009-9289-3

[mbo3365-bib-0043] Pakes, W. C. C. , and W. H. Jollyman . 1901 The collection and examination of the gases produced by bacteria from certain media. J. Chem. Soc. Trans. 79:322–329.

[mbo3365-bib-0044] Peters, J. W. , G. J. Schut , E. S. Boyd , D. W. Mulder , E. M. Shepard , J. B. Broderick , et al. 2015 [FeFe]‐ and [NiFe]‐hydrogenase diversity, mechanism, and maturation. Biochim. Biophys. Acta 1853:1350–1369. 25461840 10.1016/j.bbamcr.2014.11.021

[mbo3365-bib-0045] Pinske, C. , and R. G. Sawers . 2014 The importance of iron in the biosynthesis and assembly of [NiFe]‐hydrogenases. Biomol. Concepts 5:55–70. 25372742 10.1515/bmc-2014-0001

[mbo3365-bib-0046] Pinske, C. , M. Bönn , S. Krüger , U. Lindenstrauß , and R. G. Sawers . 2011a Metabolic deficiences revealed in the biotechnologically important model bacterium *Escherichia coli* BL21(DE3). PLoS One 6:e22830. 21826210 10.1371/journal.pone.0022830 PMC3149613

[mbo3365-bib-0047] Pinske, C. , S. Krüger , B. Soboh , C. Ihling , M. Kuhns , M. Braussemann , et al. 2011b Efficient electron transfer from hydrogen to benzyl viologen by the [NiFe]‐hydrogenases of *Escherichia coli* is dependent on the coexpression of the iron‐sulfur cluster‐containing small subunit. Arch. Microbiol. 193:893–903. 21717143 10.1007/s00203-011-0726-5

[mbo3365-bib-0048] Pinske, C. , M. Jaroschinsky , S. Linek , C. L. Kelly , F. Sargent , and R. G. Sawers . 2015 Physiology and bioenergetics of [NiFe]‐hydrogenase 2‐catalyzed H_2_‐consuming and H_2_‐producing reactions in *Escherichia coli* . J. Bacteriol. 197:296–306. 25368299 10.1128/JB.02335-14 PMC4272588

[mbo3365-bib-0050] Ripple, M. O. , N. Kim , and R. Springett . 2013 Mammalian complex I pumps 4 protons per 2 electrons at high and physiological proton motive force in living cells. J. Biol. Chem. 288:5374–5380. 23306206 10.1074/jbc.M112.438945 PMC3581419

[mbo3365-bib-0051] Rittmann, S. , and C. Herwig . 2012 A comprehensive and quantitative review of dark fermentative biohydrogen production. Microb. Cell Fact. 11:115. 22925149 10.1186/1475-2859-11-115 PMC3443015

[mbo3365-bib-0052] Romano, A. H. , and T. Conway . 1996 Evolution of carbohydrate metabolic pathways. Res. Microbiol. 147:448–455. 9084754 10.1016/0923-2508(96)83998-2

[mbo3365-bib-0053] Rossmann, R. , R. G. Sawers , and A. Böck . 1991 Mechanism of regulation of the formate‐hydrogenlyase pathway by oxygen, nitrate, and pH: definition of the formate regulon. Mol. Microbiol. 5:2807–2814. 1779767 10.1111/j.1365-2958.1991.tb01989.x

[mbo3365-bib-0054] Sapra, R. , K. Bagramyan , and M. W. W. Adams . 2003 A simple energy‐conserving system: proton reduction coupled to proton translocation. Proc. Natl Acad. Sci. USA 100:7545–7550. 12792025 10.1073/pnas.1331436100 PMC164623

[mbo3365-bib-0055] Sargent, F. , N. R. Stanley , B. C. Berks , and T. Palmer . 1999 Sec‐independent protein translocation in *Escherichia coli*. A distinct and pivotal role for the TatB protein. J. Biol. Chem. 274:36073–36082. 10593889 10.1074/jbc.274.51.36073

[mbo3365-bib-0056] Sauter, M. , R. Böhm , and A. Böck . 1992 Mutational analysis of the operon (hyc) determining hydrogenase 3 formation in *Escherichia coli* . Mol. Microbiol. 6:1523–1532. 1625581 10.1111/j.1365-2958.1992.tb00873.x

[mbo3365-bib-0057] Sawers, R. G. 1994 The hydrogenases and formate dehydrogenases of *Escherichia coli* . Antonie Van Leeuwenhoek 66:57–88. 7747941 10.1007/BF00871633

[mbo3365-bib-0058] Sawers, G. , and G. Watson . 1998 A glycyl radical solution: oxygen‐dependent interconversion of pyruvate formate‐lyase. Mol. Microbiol. 29:945–954. 9767563 10.1046/j.1365-2958.1998.00941.x

[mbo3365-bib-0059] Sawers, R. G. , S. Ballantine , and D. Boxer . 1985 Differential expression of hydrogenase isoenzymes in *Escherichia coli* K‐12: evidence for a third isoenzyme. J. Bacteriol. 164:1324–1331. 3905769 10.1128/jb.164.3.1324-1331.1985 PMC219333

[mbo3365-bib-0060] Schlindwein, C. , G. Giordano , C. L. Santini , and M. A. Mandrand . 1990 Identification and expression of the *Escherichia coli fdhD* and *fdhE* genes, which are involved in the formation of respiratory formate dehydrogenase. J. Bacteriol. 172:6112–6121. 2170340 10.1128/jb.172.10.6112-6121.1990 PMC526937

[mbo3365-bib-0061] Schuchmann, K. , and V. Müller . 2013 Direct and reversible hydrogenation of CO_2_ to formate by a bacterial carbon dioxide reductase. Science 342:1382–1385. 24337298 10.1126/science.1244758

[mbo3365-bib-0062] Stephenson, M. , and L. Stickland . 1931 Hydrogenase: a bacterial enzyme activating molecular hydrogen. I. The properties of the enzyme. Biochem. J. 25:205–214. 16744569 10.1042/bj0250205 PMC1260629

[mbo3365-bib-0063] Stephenson, M. , and L. H. Stickland . 1932 Hydrogenlyases: bacterial enzymes liberating molecular hydrogen. Biochem. J. 26:712–724. 16744879 10.1042/bj0260712 PMC1260964

[mbo3365-bib-0064] Suppmann, B. , and R. G. Sawers . 1994 Isolation and characterization of hypophosphite–resistant mutants of *Escherichia coli*: identification of the FocA protein, encoded by the pfl operon, as a putative formate transporter. Mol. Microbiol. 11:965–982. 8022272 10.1111/j.1365-2958.1994.tb00375.x

[mbo3365-bib-0065] Thauer, R. K. , K. Jungermann , and K. Decker . 1977 Energy conservation in chemotrophic anaerobic bacteria. Bacteriol. Rev. 41:100–180. 860983 10.1128/br.41.1.100-180.1977 PMC413997

[mbo3365-bib-0066] Towbin, H. , T. Staehelin , and J. Gordon . 1979 Electrophoretic transfer of proteins from polyacrylamide gels to nitrocellulose sheets: procedure and some applications. Proc. Natl Acad. Sci. USA 76:4350–4354. 388439 10.1073/pnas.76.9.4350 PMC411572

[mbo3365-bib-0067] Trchounian, A. , and R. G. Sawers . 2014 Novel insights into the bioenergetics of mixed‐acid fermentation: can hydrogen and proton cycles combine to help maintain a proton motive force? IUBMB Life 66:1–7. 24501007 10.1002/iub.1236

[mbo3365-bib-0068] Trchounian, A. A. , K. A. Bagramyan , A. V. Vassilian , and A. A. Poladian . 2000 Relationship between formate hydrogen lyase and proton‐potassium pump under heterolactic fermentation in *Escherichia coli*: functional multienzyme associations in the cell membrane. Membr. Cell Biol. 13:511–526. 10926369

[mbo3365-bib-0069] Vignais, P. M. , B. Billoud , and J. Meyer . 2001 Classification and phylogeny of hydrogenases. FEMS Microbiol. Rev. 25:455–501. 11524134 10.1111/j.1574-6976.2001.tb00587.x

[mbo3365-bib-0070] Wang, Y. , Y. Huang , J. Wang , C. Cheng , W. Huang , P. Lu , et al. 2009 Structure of the formate transporter FocA reveals a pentameric aquaporin‐like channel. Nature 462:467–472. 19940917 10.1038/nature08610

[mbo3365-bib-0071] Weiss, H. , T. Friedrich , G. Hofhaus , and D. Preis . 1991 The respiratory‐chain NADH dehydrogenase (complex I) of mitochondria. Eur. J. Biochem. 197:563–576. 2029890 10.1111/j.1432-1033.1991.tb15945.x

[mbo3365-bib-0072] Woods, D. D. 1936 Hydrogenlyases: the synthesis of formic acid by bacteria. Biochem. J. 30:515–527. 16746050 10.1042/bj0300515 PMC1263052

[mbo3365-bib-0073] Wu, L. F. , and M. A. Mandrand‐Berthelot . 1993 Microbial hydrogenases: primary structure, classification, signatures and phylogeny. FEMS Microbiol. Rev. 10:243–269. 8318259 10.1111/j.1574-6968.1993.tb05870.x

[mbo3365-bib-0074] Wyborn, N. R. , S. L. Messenger , R. A. Henderson , R. G. Sawers , R. E. Roberts , M. M. Attwood , et al. 2002 Expression of the *Escherichia coli yfiD* gene responds to intracellular pH and reduces the accumulation of acidic metabolic end products. Microbiology 148:1015–1026. 11932447 10.1099/00221287-148-4-1015

[mbo3365-bib-0075] Zhu, S. , and S. B. Vik . 2015 Constraining the lateral helix of respiratory complex I by cross‐linking does not impair enzyme activity or proton translocation. J. Biol. Chem. 290:20761–20773. 26134569 10.1074/jbc.M115.660381 PMC4543639

[mbo3365-bib-0076] Zickermann, V. , C. Wirth , H. Nasiri , K. Siegmund , H. Schwalbe , C. Hunte , et al. 2015 Structural biology. Mechanistic insight from the crystal structure of mitochondrial complex I. Science 347:44–49. 25554780 10.1126/science.1259859

