# Supplementary material for: Exploring the directionality of Escherichia coli formate hydrogenlyase: a membrane‐bound enzyme capable of fixing carbon dioxide to organic acid
Source: Microbiologyopen. 2016 May 2;5(5):721–37. doi: 10.1002/mbo3.365 (PMC5061711; doi:10.1002/mbo3.365)
Supplement: Supplementary file 1 — Figure S1. Analysis of downstream polar effects. Figure S2. Inhibition of H2 production through addition of protonophore CCCP (Carbonyl cyanide m‐chlorophenyl hydrazone). Table S1. Oligonucleotides used in this work. [file MBO3-5-721-s001.docx]

**Supplementary Information for:**

**Exploring the directionality of *Escherichia coli* formate hydrogenlyase: a membrane-bound enzyme capable of fixing carbon dioxide to organic acid.**

Constanze Pinske^a^ and Frank Sargent*

Division of Molecular Microbiology, School of Life Sciences, University of Dundee, Dundee DD1 5EH, Scotland, UK.

^a^present address: Martin-Luther University Halle-Wittenberg, Institute of Biology/Microbiology, Kurt-Mothes-Str. 3, 06120 Halle (Saale), Germany

**Corresponding author:** Prof Frank Sargent, Division of Molecular Microbiology, School of Life Sciences, University of Dundee, Dow Street, Dundee DD1 5EH, Scotland, United Kingdom. E: [f.sargent@dundee.ac.uk](mailto:f.sargent@dundee.ac.uk), T: +44 (0)1382 386 463, F: +44 (0)1382 388 216

**
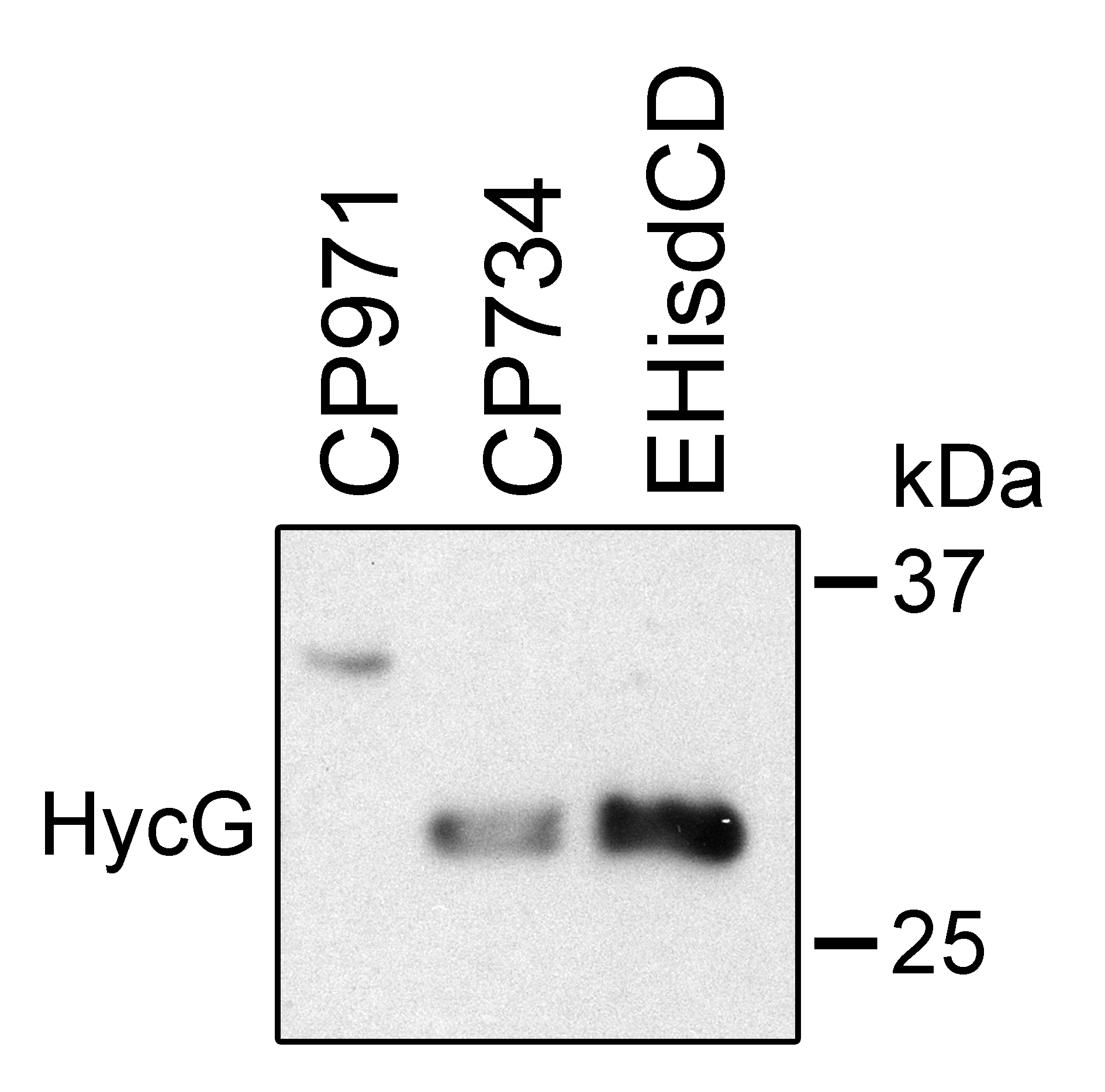
**

**Figure S1: Analysis of downstream polar effects.** Western blot analysis of crude extracts derived from strains CP971 (Δ*hycAI*), CP734 (Δ*hyaB hybC*) and EhisdCD for the presence of HycG polypeptide. An amount of 25 µg of protein from crude extracts of anaerobically grown cells was applied to 10% (w/v acrylamide) SDS-PAGE, separated, transferred to nitrocellulose, and challanged with antibodies raised against HycG as described in Material and Methods. The migration of the ladder is given on the right hand side and the migration of the HycG polypeptide is indicated on the left.


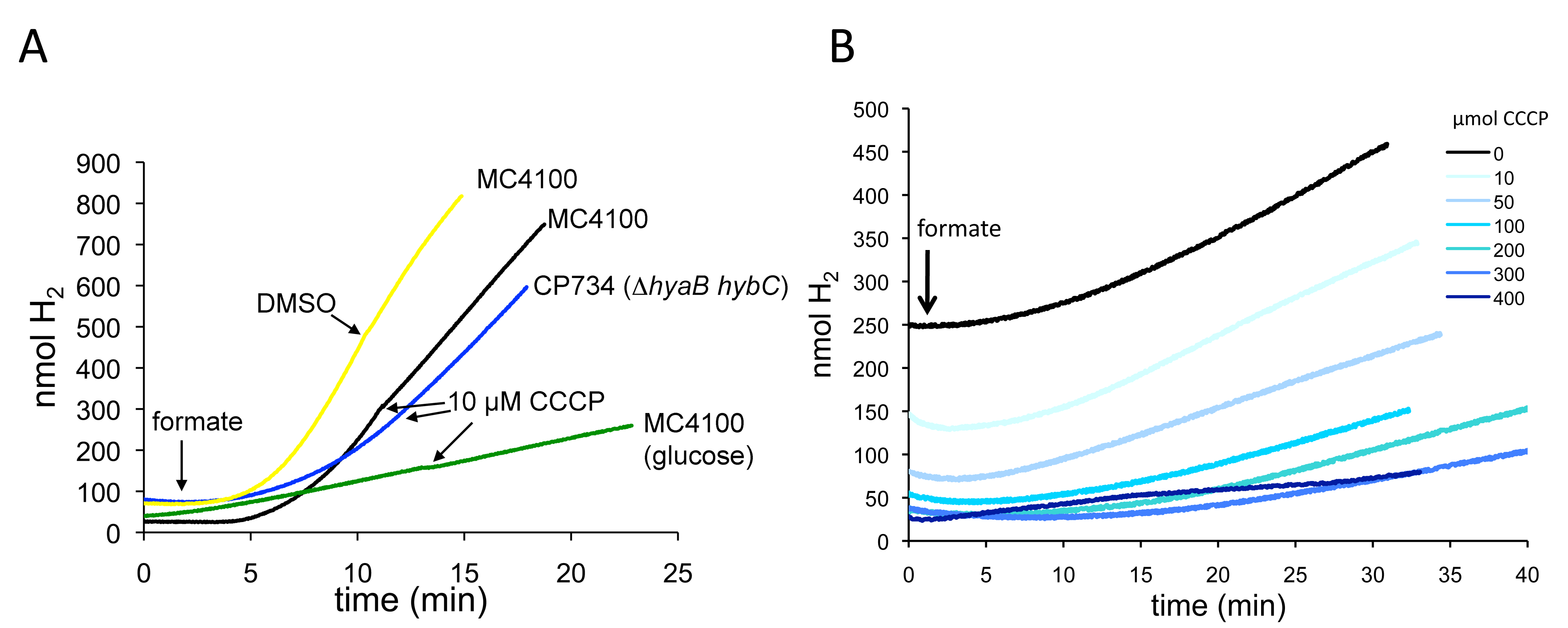


**Figure S2: Inhibition of H_2_ production through addition of protonophore CCCP (Carbonyl cyanide *m*-chlorophenyl hydrazone)**. A modified Clark-type electrode was filled with 50 mM MOPS, pH 7.0 and 100 µl cell suspension of either MC4100 or CP734 (Δ*hyaB hybC*, blue trace) corresponding to 6.8 mg protein was added as indicated and the reaction started through the addition of 17 mM formate (arrow). When the reaction reached linearity CCCP was added to a final concentration of 10 µM as indicated and the H_2_ production was further followed. Alternatively the reaction was started with glucose (green trace) or DMSO only was added (yellow trace). **(B)** CCCP was added to the reaction chamber in the indicated concentrations and the reaction started with 15 mM formate. All reactions were carried out in the presence of identical volumes of ethanol, the CCCP solvent.

**Table S1: Nucleotides used**

| Exchange in HycC | Primers used (5’ 🡪 3’) |
| --- | --- |
| T83A | TTGGCTGATTgCGCTCGGTCT |
|  | ATCGCGTTAAGCGGAGAG |
| E135A | GTGGTAATGGCCGcAATCATGGCCCTG |
|  | CAGGGCCATGATTgCGGCCATTACCAC |
| L208K | CTTTGGCCTGaaGGCCGGGATTATT |
|  | CCAATCACTCCGAGCAGC |
| H215A | TATTCCGCTGgcCGGCTGGGTGCCGCAGG |
|  | ATCCCGGCCAGCAGGCCA |
| H222A | GCCGCAGGCAgcTGCGAACGCCTCTGCG |
|  | ACCCAGCCGTGCAGCGGA |
| K239A | GGTAGTCATGgcAATTGGCCTGC |
|  | GTAGAAAACAACGCGGCAG |
| E281A | GTATGCGCTGGTGGcaCACAACATCCAGC |
|  | GCTGGATGTTGTGtgCCACCAGCGCATAC |
| T292A | GGCTTACCACgCCCTGGAAAATATC |
|  | AGCAGGCGCTGGATGTTG |
| E294A | CTTACCACACCCTGGcAAATATCGGCATCATC |
|  | GATGATGCCGATATTTgCCAGGGTGTGGTAAG |
| N295A | CACCCTGGAAgcTATCGGCATCATCCTGCTG |
|  | TGGTAAGCCAGCAGGCGC |
| H328A | GGTGGTCTGTACgcTCTGCTTAACCAT |
|  | ATGGTTAAGCAGAgcGTACAGACCACC |
| H332A | CATCTGCTTAACgcTAGCCTGTTCAAA |
|  | TTTGAACAGGCTAgcGTTAAGCAGATG |
| K336A | CATAGCCTGTTCgcAAGCGTACTGTTC |
|  | GAACAGTACGCTTgcGAACAGGCTATG |
| D354A | GGTCATCGCGcTATCGAAAAACTC |
|  | GGTACGGAACCAGACGCT |
| N386A | GCCGCCGCTGgcTGGTTTTGCC |
|  | AGCGCAGCCATTGCCATC |
| F388A | GCTGAATGGTgcTGCCGGGGAATGGGTTATC |
|  | GGCGGCAGCGCAGCCATT |
| E391A | GGTTTTGCCGGGGcATGGGTTATCTATC |
|  | GATAGATAACCCATgCCCCGGCAAAACC |
| Exchange in HycD |  |
| E138A | CGTGCTGGTCGcACCGATGCTGC |
|  | CCAAGCATCGCTTCACGGC |
| E189A | CACCTTTATCGcAATGGGCAAACTGC |
|  | GCGAACGCACAGGCACAA |
| E201A | GGCGGAAGCCGcGCAGGAGCTGC |
|  | AGGTCGAACGGCAGTTTGCCC |
| E203A | AGCCGAGCAGGcGCTGCAGGAAG |
|  | TCCGCCAGGTCGAACGGC |
| E199/201/203A | GCAGGcGCTGCAGGAAGGCCCGCTC |
|  | gCGGCagCCGCCAGGTCGAACGGCAG |
| Various oligonucleotides |  |
| hycB -500FW | GCG AAGCTT CCTGAGAGTGATGCTGAATG |
| hycB end | GCG GGATCC TCATTTAGCCTCTCCACTTTG |
| hycE start | GCG GGATCC TTAAAGAGAGTTTGAGCATG |
| hycE +500RW | GCG AAGCTT CGGTGCCGGACGCTGACGAT |

*underlined nucleotides represent the respective codon and small letters the exchanged residues
